# Metabolic reprogramming in hepatocellular carcinoma: a bibliometric and visualized study from 2011 to 2023

**DOI:** 10.3389/fphar.2024.1392241

**Published:** 2024-07-16

**Authors:** Xia Li, Liping Zhou, Xinyi Xu, Xiyang Liu, Wenjun Wu, Quansheng Feng, Ziwei Tang

**Affiliations:** ^1^ School of Basic Medical Sciences, Chengdu University of Traditional Chinese Medicine, Chengdu, China; ^2^ The Beibei Affiliated Hospital of Chongqing Medical University, The Ninth People’s Hospital of Chongqing, Chongqing, China

**Keywords:** hepatocellular carcinoma, metabolic reprogramming, tumor microenvironment, Warburg effect, bibliometric

## Abstract

**Background and aims:**

Metabolic reprogramming has been found to be a typical feature of tumors. Hepatocellular carcinoma (HCC), a cancer with high morbidity and mortality, has been extensively studied for its metabolic reprogramming-related mechanisms. Our study aims to identify the hotspots and frontiers of metabolic reprogramming research in HCC and to provide guidance for future scientific research and decision-making in HCC metabolism.

**Methods:**

Relevant studies on the metabolic reprogramming of HCC were derived from the Web of Science Core Collection (WoSCC) database up until November 2023. The bibliometrix tools in R were used for scientometric analysis and visualization.

**Results:**

From 2011 to 2023, a total of 575 publications were obtained from WoSCC that met the established criteria. These publications involved 3,904 researchers and 948 organizations in 37 countries, with an average annual growth rate of 39.11% in research. These studies were published in 233 journals, with *Cancers* (*n* = 29) ranking first, followed by *Frontiers in Oncology* (*n* = 20) and *International Journal of Molecular Sciences* (*n* = 19). The top ten journals accounted for 26% of the 575 studies. The most prolific authors were Wang J (*n* = 14), Li Y (*n* = 12), and Liu J (*n* = 12). The country with the most publications is China, followed by the United States, Italy, and France. Fudan University had the largest percentage of research results with 15.48% (*n* = 89). Ally A’s paper in *Cell* has the most citations. A total of 1,204 keywords were analyzed, with the trend themes such as “glycolysis,” “tumor microenvironment,” “Warburg effect,” “mitochondria,” “hypoxia ,” etc. Co-occurrence network and cluster analysis revealed the relationships between keywords, authors, publications, and journals. Moreover, the close collaboration between countries in this field was elucidated.

**Conclusion:**

This bibliometric and visual analysis delves into studies related to metabolic reprogramming in HCC between 2012 and 2023, elucidating the characteristics of research in this field, which has gradually moved away from single glycolipid metabolism studies to the integration of overall metabolism in the body, pointing out the trend of research topics, and the dynamics of the interaction between the tumor microenvironment and metabolic reprogramming will be the future direction of research, which provides blueprints and inspirations for HCC prevention and treatment programs to the researchers in this field.

Systematic Review Registration: [https://www.bibliometrix.org].

## 1 Introduction

Today, cancer remains the leading cause of death among the world’s population. Liver cancer ranks sixth in incidence and third in mortality among cancers in the Global Cancer Database 2020, underscoring its high degree of malignancy (IARC, 2020). More than 90% of liver cancer cases are hepatocellular carcinoma (HCC) (Llovet et al., 2016). Compared to the world ranking, HCC morbidity and mortality rates in Asian countries have increased by one place, respectively (Zhang et al., 2022). It is estimated that around 50% of the global population of patients with HCC are from China (Llovet et al., 2021). This indicates that HCC has become a significant global challenge, with China being particularly affected (Villanueva, 2019). Although hepatitis B and C viruses and alcohol remain the primary causes of HCC, non-alcoholic fatty liver disease (NAFLD) associated with metabolic dysregulation is gradually becoming a crucial risk factor for HCC, and has been receiving more attention in recent years (Estes et al., 2018). Moreover, since NAFLD involves abnormalities in fat and glucose metabolism, often resulting in conditions such as obesity or diabetes, NAFLD-associated HCC has a unique pathogenesis involving metabolic, oxidative stress, pathological inflammatory response, and altered immune function (Anstee et al., 2019). To draw attention to metabolic abnormalities, academics have collectively referred to liver disease associated with obesity, diabetes, and systemic metabolic abnormalities as metabolic dysfunction-associated fatty liver disease (MAFLD), replacing the terminology NAFLD and “non-viral” (Eslam et al., 2020). With lifestyle changes, MAFLD may be a major contributor to liver cancer in the future (Huang et al., 2021). MAFLD-associated HCC is more likely to have metabolic complications (Tan et al., 2022), tends to have larger tumors, and more often than not does not go through the cirrhotic stage, leaving a high proportion of patients with a lack of indications for surveillance (Crane et al., 2023). HCC can be managed through various strategies such as surgery (resection, liver transplantation), local regional (ablation and embolization therapy), and pharmacological treatment. However, the current situation is not very optimistic. The development of metabolically targeted drugs such as 2-Deoxy-d-glucose (noncompetitively inhibit the activity of HK2), TKT inhibitor oxythiamine (thiamine antagonist), and TVB-2640 (fatty acid synthase inhibitor) is still in the early stage and many are still in preclinical studies (Du et al., 2022). A comprehensive understanding of the pathological mechanisms of HCC is necessary to develop targeted therapeutic strategies to improve the poor prognosis of HCC.

Metabolic reprogramming refers to the process of systematic adjustment and transformation of the metabolic pattern of a cell to adapt to changes in the external environment and to meet the needs of its own growth and differentiation under specific physiological and pathological conditions, which involves a fundamental change in the way the cell acquires and uses energy and raw materials for biosynthesis, and involves the regulation of a number of metabolic pathways, including glycolysis, oxidative phosphorylation (OXPHOS), fatty acid metabolism, amino acid metabolism, and so on. Cells rely on metabolism to produce energy for their survival and function, with normal human cells using mitochondrial OXPHOS primarily under aerobic conditions and turning to glycolysis when oxygen is scarce. However, tumor cells differ from normal cells in that they prefer cytoplasmic glycolysis even when oxygen is available. The “Warburg effect” or “aerobic glycolysis” was first identified by Otto Warburg in 1956 (Warburg, 1956), and is attributed to the uncontrolled proliferation of tumors and activation of invasive and metastatic pathways. Tumor cells require large amounts of ATP and biomaterials for high biomass synthesis, which can lead to hypoxia and nutrient deficiencies (Martínez-Reyes and Chandel, 2021). Cancer cells undergo biological changes to fulfill their high energy demands as they evolve. Studies have shown that the catabolism and anabolism of cancer cells are greatly enhanced, with genes related to glycolysis, the pentose phosphate pathway (PPP), nucleotide biosynthesis, the tricarboxylic acid cycle, and oxidative phosphorylation persistently upregulated while genes for xenobiotic, fatty acids, and amino acid metabolism are downregulated (Nwosu et al., 2017). These metabolic alterations in HCC have been consistently associated with the Warburg effect. Another study also revealed that major metabolic changes in HCC include upregulation of glycolysis, gluconeogenesis, and β-oxidation as well as downregulation of the tricarboxylic acid (TCA) cycle (Huang et al., 2013). Glycolytic enzymes such as Glut1, Glut4, and HK2 are often elevated in HCC and have been linked to poor prognosis for patients (Schwartzenberg-Bar-Yoseph et al., 2004; Amann et al., 2009; DeWaal et al., 2018; Chai et al., 2019). Glucose-6-phosphate dehydrogenase (G6PD) is a crucial enzyme that regulates the PPP. Clinical studies have found that the expression level of G6PD in tumor tissues of HCC is significant, in comparison to adjacent normal tissues. Moreover, metastatic HCC tissues exhibited a higher level of G6PD compared to non-metastatic tissues, which is directly correlated with shorter survival (Lu et al., 2018). Increased glutamine metabolism is also a key feature of altered tumor metabolism, which produces higher levels of α-ketoglutarate and citrate to support the mitochondrial TCA cycle. In addition, the aberrant lipid metabolism in HCC also plays an important role in carcinogenesis. Notably, HCC cells typically exhibit higher rates of lipid de novo synthesis and fatty acid β-oxidation (FAO) uptake. The significance of glycolipid metabolic reprogramming in all aspects of hepatocarcinogenesis and development is increasingly supported by a growing body of evidence. Therefore, this study will focus on recent findings of metabolic reprogramming in HCC.

Bibliometrics is the cross-cutting science that quantitatively analyzes all knowledge carriers using mathematical and statistical methods. This comprehensive field combines mathematics, statistics, and documentation to quantify and analyze relationships between published works based on abstracts, keywords, citations, and other relevant information (Ninkov et al., 2022). Bibliometrics is an important tool that can be used to understand how a particular topic has been researched in the literature, track the research trajectory of the topic, and identify the characteristics of high-impact journal articles. Since the formalization of bibliometric research, more and more researchers have carried out important work using journal impact factors, H-indexes, and visualization methods to identify the structure of knowledge, current developments, and research frontiers in specific fields (Hassan et al., 2021). Bibliometric analysis is a quick way to identify the most representative authors, institutions, countries, and journals in a particular field of study (Wang and Maniruzzaman, 2022). A software package called biblioshiny, which operates in an R environment, is a typical bibliometric research software that allows for a friendly graphical interface to help researchers quickly understand the scientific field. Its interactive visual user interface makes it easy to use (Khan et al., 2021), and it integrates bibliographic analysis and visualization into one package. Biblioshiny offers high convenience and flexibility, providing plotting under multiple analyses and customization of different types of advanced charts. This significantly improves the efficiency of the researchers’ work compared to traditional analysis software.

In recent years, the study of cancer metabolism has been gradually emerging thanks to the development of systems biology and extensive research. This has led to an increase in publications on the topic, particularly about metabolic reprogramming-based studies on the pathomechanisms and action targets in cancer. In this context, we conducted a bibliometric analysis of publications on metabolic reprogramming in HCC over the past 12 years using biblioshiny. We aimed to provide insights into the current status of metabolic reprogramming in HCC research and future research trends. We hope that this comprehensive bibliometric analysis will help researchers in this field to conduct more systematic and targeted explorations.

## 2 Materials and methods

### 2.1 Literature extraction

This study quantitatively assessed existing scientific results on metabolism related to HCC to characterize the evolution of HCC metabolism research over the last decade. In the current work, we conducted a literature search on November 13, 2023, using the search terms “hepatocellular carcinoma” and “metabolic reprogramming” to retrieve literature published between 2011 and 2023 from the Web of Science Core Collection (WoSCC) Science Citation Index Expanded (SCIE), a mainstream and authoritative high-quality database of all types of materials. We limited the type of publication to “articles” and “reviews,” restricted the language to English to ensure the representativeness of the included studies, and exported “fully documented and cited references.” Two independent reviewers were involved in the process and excluded duplicate/unrelated documents to ensure the accuracy of the scientometric analysis. Data collected included title, author, institution, country, journal, abstract, keywords, and references. If there was a disagreement between two reviewers, it was resolved through joint negotiation by a third independent reviewer.

### 2.2 Statistical and visual analysis

We used the R language package Bibliometrix (Aria and Cuccurullo, 2017) for the scientometric analysis and visualization. Bibliometrix provides all the tools for a complete bibliometric analysis following a scientific mapping workflow. The package was built in R, a programming language for statistical computing and graphics. In this study, the basic information obtained about the metabolic research field of HCC was uploaded to Bibliometrix, and analyzed by automatic algorithms and machine intelligence for country, journal, author productivity, and institution. We mainly used two major series of functions of Bibliometrix, adopting bibliometrics-based analysis and extraction techniques for analyzing indicators and mining techniques for literature-related concepts, knowledge, and social structures for correlation analysis. Firstly, we use clean corpus() function to clean the literature data, remove invalid information or incorrectly formatted data, and extract the metadata information of the literature data such as authors, journals, etc., to lay the foundation for the subsequent analysis work, and then use biblioNetwork() function to construct the literature co-citation graph, and use biblioAnalysis() function to conduct the literature research trend analysis, in-depth understanding of the analyzed literature data. In addition, thematic modeling analysis, temporal analysis, network analysis, MCA, and clustering techniques were performed to analyze the realized network matrix and historical network matrix, to discover the hidden patterns and trends in the literature data. Finally, networkPlot(), histPlot(), and conceptualStructure() functions were used for network visualization and conceptual structure visualization.

## 3 Results

### 3.1 Basic characteristics of the publication

**FIGURE 1:**
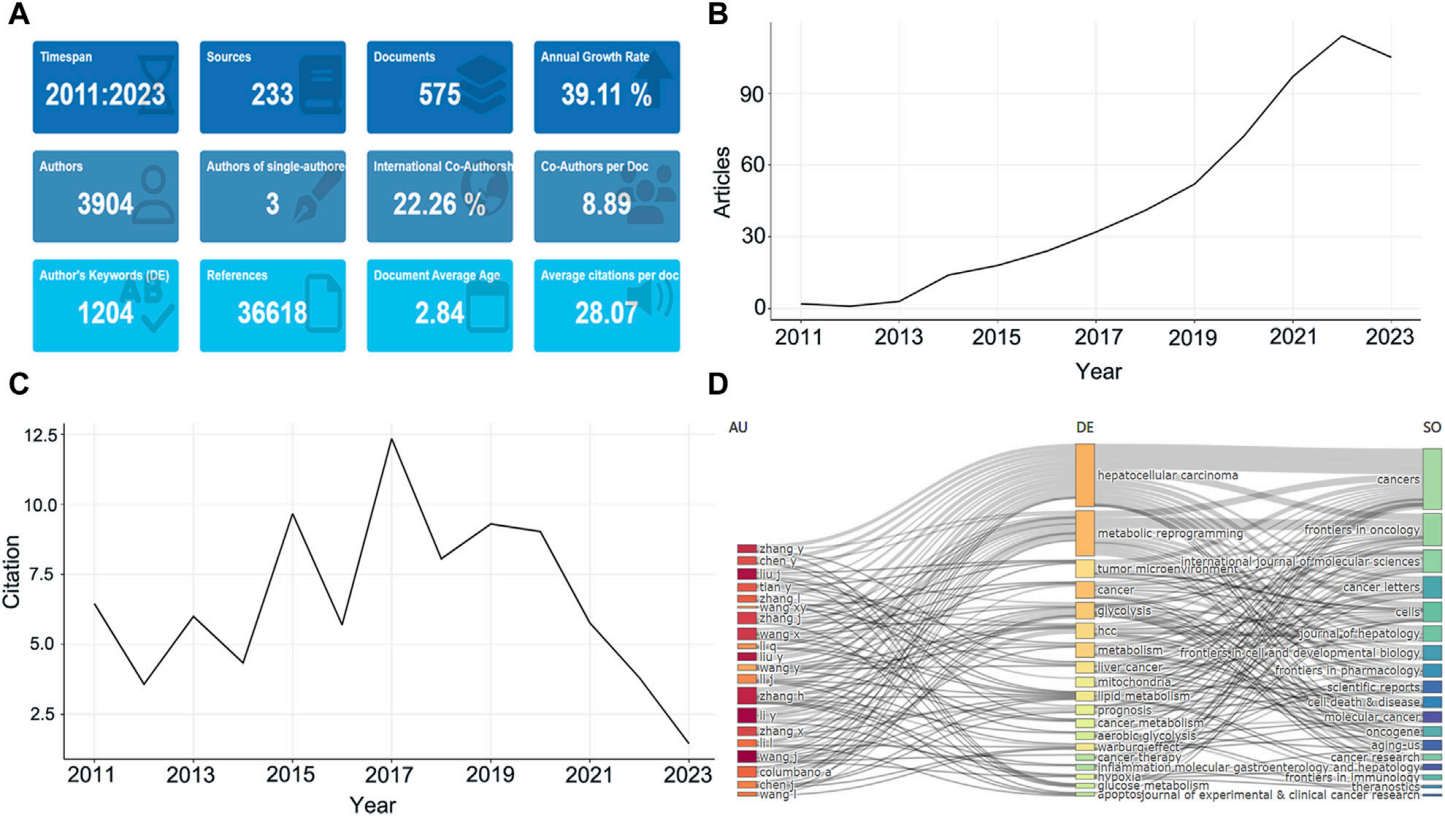
The basic features of published studies. **(A)** The main information of publications. **(B)** Annual growth of the documents. **(C)** Average citations per year of documents. **(D)** Three-Fields Plot. The left is the author, the middle is keywords, right is journal sources.

A total of 575 studies meeting the eligibility criteria were collected at WoSCC, involving 3,904 authors (Figure 1A). Only three documents were single-authored, while the rest contained multiple authors, with an average of 8.89 authors per document, and international collaborations accounting for 22.26% of the documents (Figure 1A). Literature published in this field has been increasing year by year from 2011–2023 with an annual growth rate of 39.11% (Figure 1B). This peaked in 2022 with 114 publications, or 19.8% of the total, with a slight drop-off in 2023, probably related to the fact that the amount of data for the whole year was not counted. All documents have been cited in 36,618 references while the average number of citations per document is 28.07 (Figure 1A). The average citations per year from 2011 to 2023 in these documents is also shown in Supplementary Table S1, with articles from 2015 to 2019 being cited the most, indicating that the literature in this period has a greater impact (Figure 1C). Additionally, a three-fields plot of authors, keywords, and publication sources for the top twenty most relevant documents in this research area was developed (Figure 1D).

### 3.2 Analysis of representative journals

**FIGURE 2:**
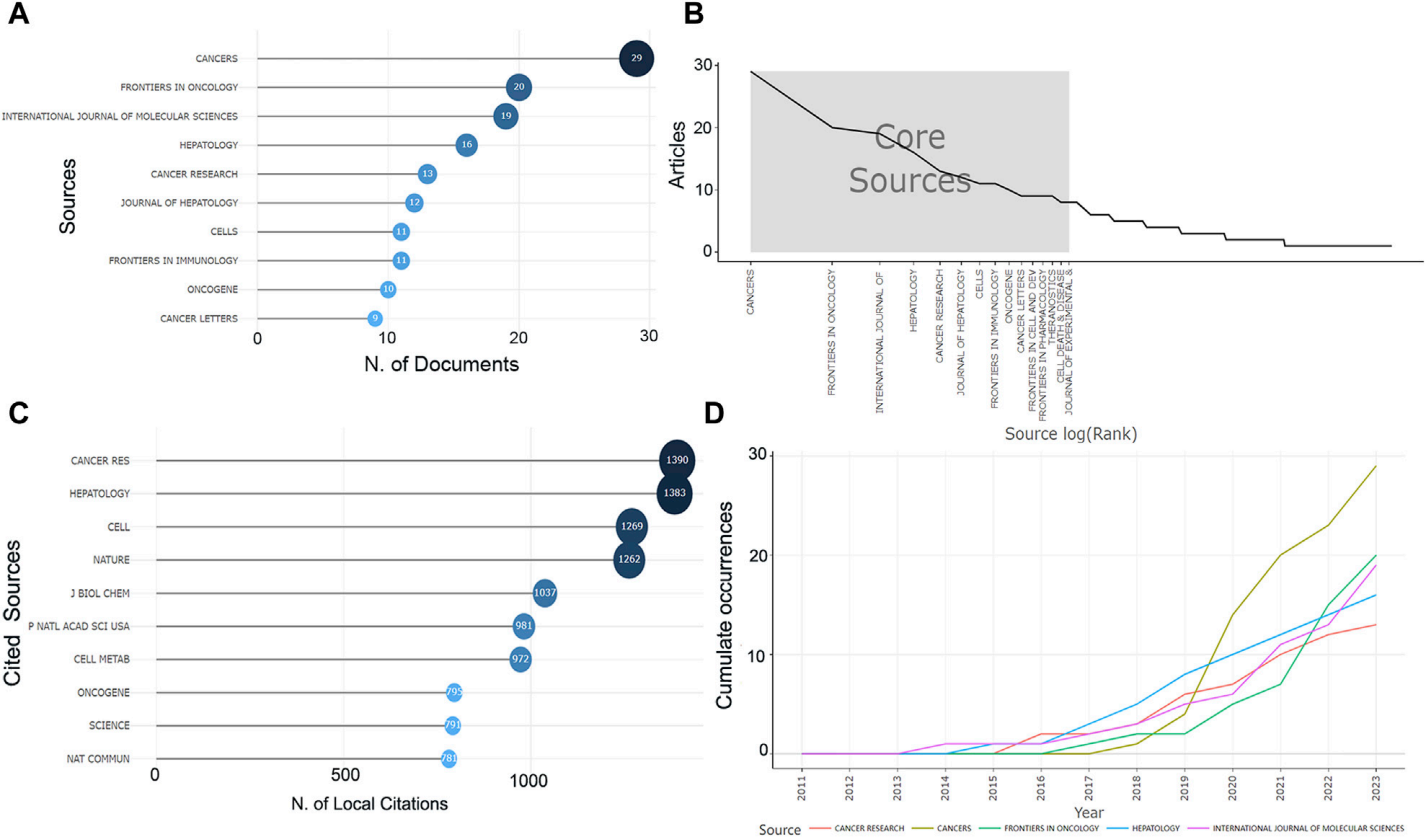
Information about the journal. **(A)** The number of publications in the journal. **(B)** The core journals. **(C)** The number of citations of journals. **(D)** The cumulative number of publications in recent years in the top five journals in this field.

These studies were originated from 233 publications. The most published journal in this research area is *Cancers* with 29 articles, followed by *Frontiers in Oncology* (*n* = 20), *International Journal of Molecular Sciences* (*n* = 19) and *Hepatology* (*n* = 16) (Figure 2A). The top ten journals collectively published about 26% of the articles and were derived by Bradford’s Law as the core regional journals in the field (Figure 2B). Among the top ten journals it can be observed that the vast majority are high impact journals with impact factors (IF) ranging from 4.7 to 25.7, and about 70% scored Q1 in the JCR division (Supplementary Table S2). *Journal of Hepatology* and *Hepatology* have high IF, while *Cancers* has the highest total citations (TCs). Moreover, among the most cited local journals, *Cancer Research* (Citations = 1,390), *Hepatology* (Citations = 1,383), and *Cell* (Citations = 1,269) ranked in the top three (Figure 2C), highlighting the high impact. The H-index represents to some extent the number of academic outputs and the level of outputs, whereas the *International Journal of Molecular Sciences* was ranked first in this research area, followed by *Cancers*, *Hepatology* (Supplementary Table S2). Finally, we have observed the cumulative output of the five most productive journals in this research area for the period from 2011 to 2023 (Figure 2D). The first journal to publish research on the topic of HCC metabolism was the *International Journal of Molecular Sciences*, the most represented journal between 2016 and 2019 was *Hepatology*, while after 2020 the production of *Cancers* skyrocketed way ahead of the other journals.

### 3.3 Analysis of authors

**FIGURE 3:**
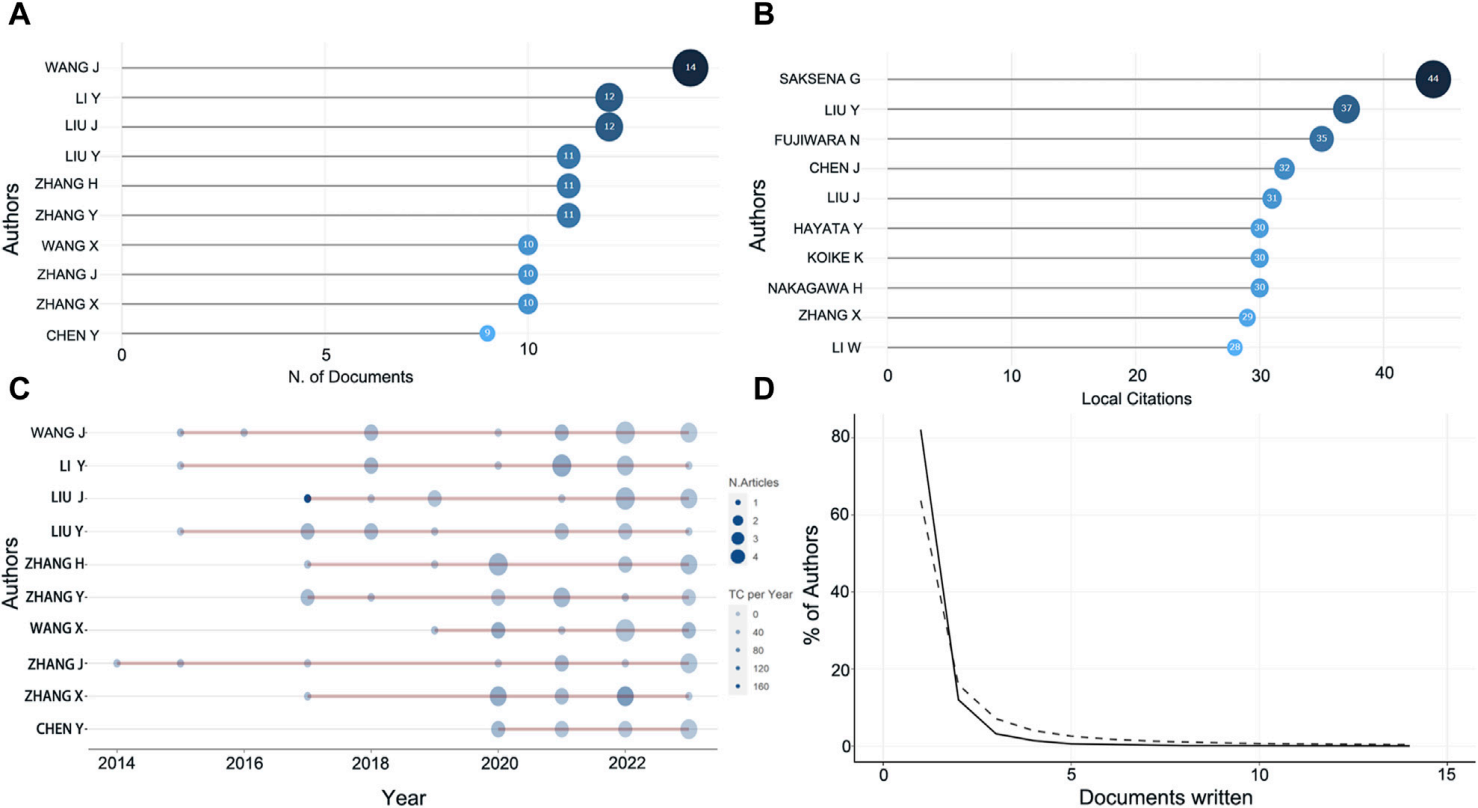
Information about the authors. **(A)** The top ten most prolific authors. **(B)** Top ten highly cited authors. **(C)** Number of publications and citations in recent years of prolific authors. Color intensity and bubble size are positively correlated with the total number of annual citations and the number of documents, respectively. **(D)** Lotka’s law.

The most prolific author in this field of research is Wang J with 14 articles involved, followed by Li Y, Liu J, and Liu Y (Figure 3A). However, the most cited authors are Saksena G, Liu Y, Fujiwara N, all with more than 35 citations (Figure 3B). To more objectively explore the authors with high academic level in this research field, we further tracked the output of these highly productive top ten authors in recent years and found that Liu J published one article in 2017 with a total citation count of 168.29, and four articles in 2022 with a total citation count of 19.5 (Figure 3C). Similarly, Li Y, Zhang H, Zhang Y, Wang X, Zhang X all published high impact articles between 2020 and 2022 with an average of more than 20 citations per year (Figure 3C). The H-index still showed that Liu J, Li Y, Liu Y et al. are still highly influential, which is consistent with the trend presented by the production and citation numbers in recent years (Table 1). It can be observed that the percentage of authors decreases as the scientific output in the field of study continues to be produced, in accordance with Lotka’s Law (Figure 3D).

**TABLE 1:** The top ten authors’ impact in this field.

Author	H index	G index	M index	TC	PY start
Liu J	9	12	1.286	1,310	2017
Li Y	8	12	0.889	372	2015
Liu Y	8	11	0.889	373	2015
Zhang H	8	11	1.143	234	2017
Zhang X	8	10	1.143	398	2017
Li L	7	8	1	114	2017
Wang J	7	14	0.778	452	2015
Wang X	7	10	1.4	254	2019
Zhang Y	7	11	1	281	2017
Chen J	6	7	0.75	1,310	2016

TC, total citations; PY, start, the year of the author’s first publication.

### 3.4 Distribution of countries and institutions

**FIGURE 4:**
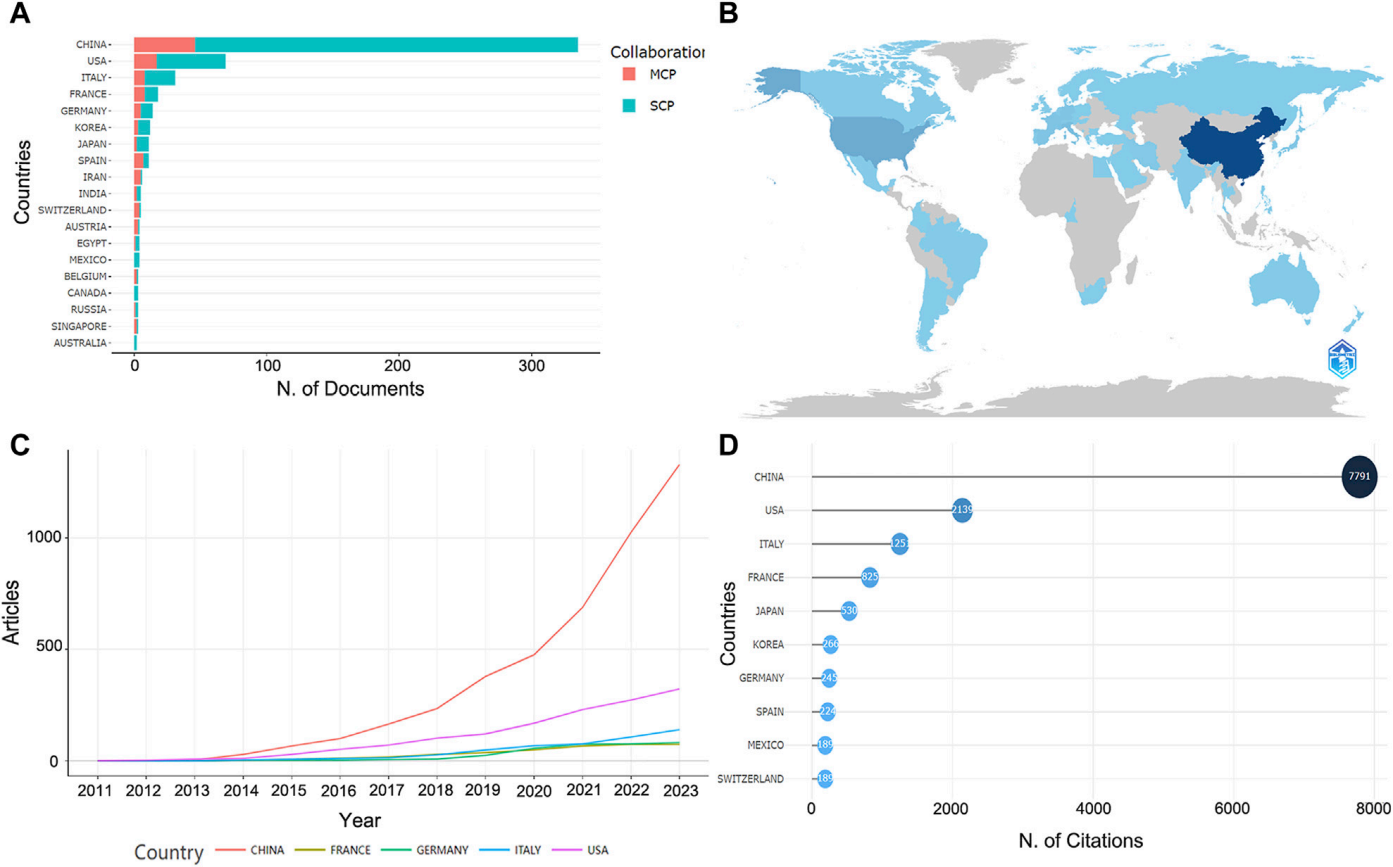
The information about countries of publications. **(A)** Corresponding author’s countries. MCP, number of papers co-authored with authors from other countries. SCP, number of papers co-authored with authors from the same country. **(B)** The number of papers in each country. The darker the color, the higher the number of papers. **(C)** Trends in the number of papers by the top five high-producing countries over time. **(D)** Highly cited countries and total citations.

Based on the corresponding authors of the manuscripts, it can be determined that a total of 37 countries are involved, with China publishing the most research (*n* = 335), accounting for 58.3% of the total, significantly more than any other country, followed by the United States (*n* = 69, 12%), Italy (*n* = 31, 5.4%), France (*n* = 18, 3.1%), and Germany (*n* = 14, 2.4%) (Figures 4A, B; Supplementary Table S3). It can be found that the number of collaborative papers with the same nationality among the countries is predominant, while Iran is the highest in terms of MCP ratio (Supplementary Table S3). The development trend of the top five countries in terms of the number of publications in recent years showed that China has always been ahead of other countries, especially after 2020, when the number of publications showed a remarkable upward trend (Figure 4C). As for the number of paper citations, China is still the highly cited country with 7,791 citations, followed by the United States (TCs = 2,139), Italy (TCs = 1,251) and France (TCs = 825) (Figure 4D). In terms of the average number of paper citations, Japan, Mexico and France, are the highest, with 48.2, 47.2, and 45.8 respectively, indicating their advanced academic level in this research field (Supplementary Table S4).

**FIGURE 5:**
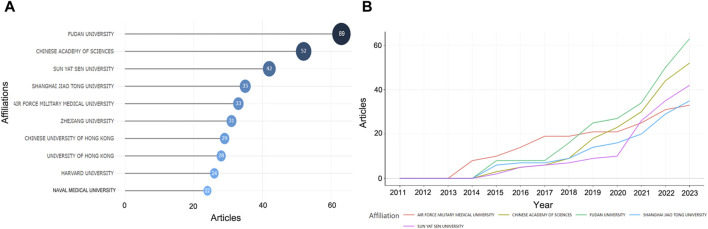
The most relevant affiliations. **(A)** The top ten affiliations. **(B)** Trends in the number of papers by the top five high-producing affiliations over time.

Figure 5A illustrates the institutions contributing to this field, involving 948 institutions, with the most published research coming from Fudan University (*n* = 89), the Chinese Academy of Sciences (*n* = 52), and Sun Yat-sen University (*n* = 42), all from China. The top five institutions accounted for 43.66% of the 575 research publications. Although the earliest published study was from the Air Force Military Medical University, it showed a slow growth trend after 2017. Fudan University, Chinese Academy of Sciences, and Sun Yat-sen University, on the other hand, have shown a rapid growth trend in the last 3 years (Figure 5B).

### 3.5 Analysis of cited references

**FIGURE 6:**
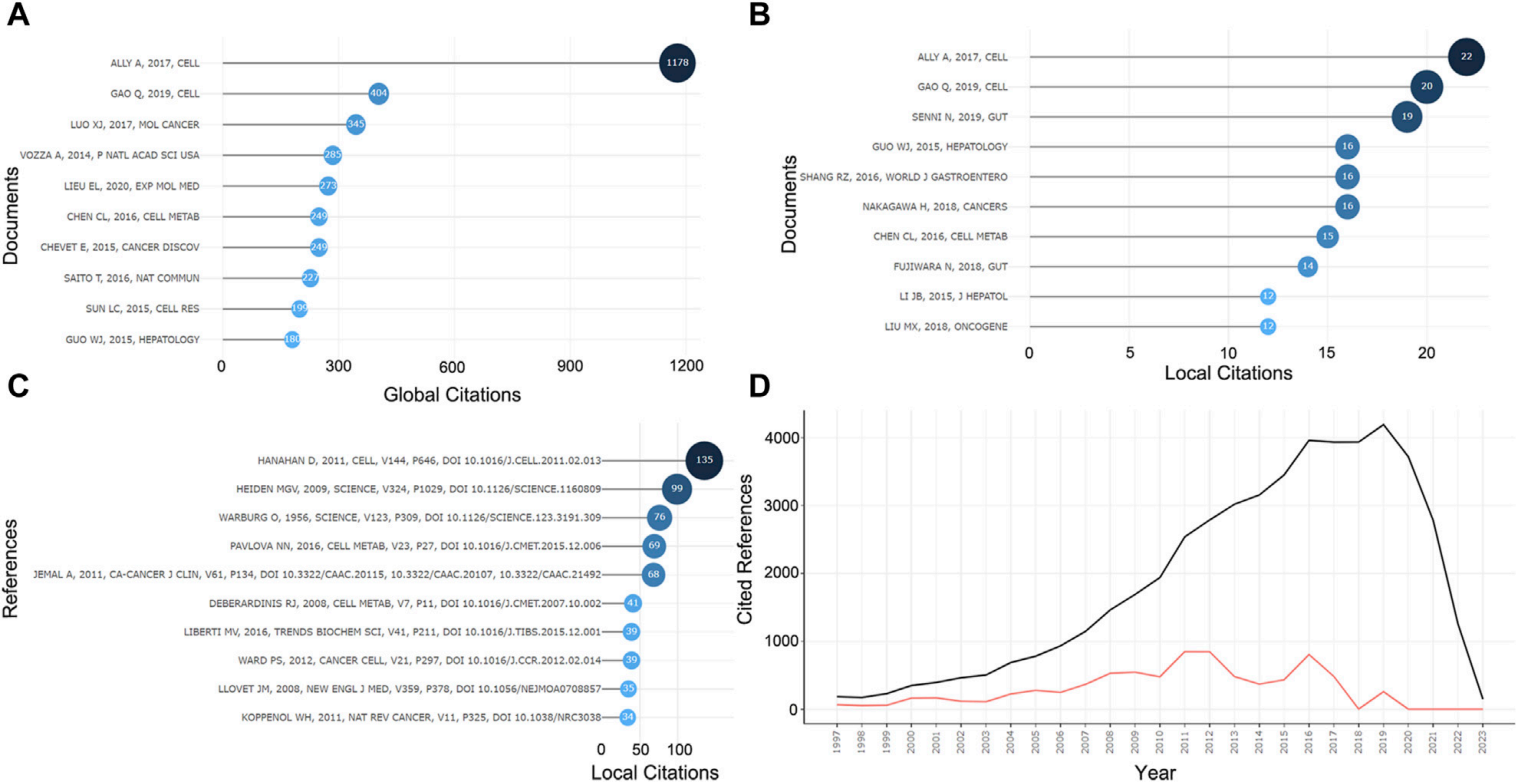
Information about cited papers and references. **(A)** Top ten highly cited papers in WOCSS in 575 papers. **(B)** Top ten highly cited papers in the current dataset. **(C)** Top ten highly cited references in the current dataset. **(D)** Number of references per year.

In the 575 publications, we focused on the highly cited literature. Ally A’s paper published in *Cell* in 2017 was the focus of attention, and it ranked first in TCs for both the global WoSCC citations and the local citations, with 1,178 and 22, respectively (Figures 6A, B). Given the high degree of relevance to the current field of research, we focused primarily on the local citations and listed the top ten articles (Table 2). LC/GC ratio could indicate the impact of this article’s results in this research area on the broader field of HCC. Among the top ten locally cited documents, Senni N’s study published in *Gut* has the greatest impact, accounting for 24.68% (Table 2). Senni et al. (2019) formally proposed PPAR-α as a target involved in the metabolism reprogramming of fatty acid oxidative in β-conjugated protein-causing HCC. In addition, we analyzed the references that were most cited by the current dataset (Figure 6C). Hanahan D’s review article (Hanahan and Weinberg, 2011) in *Cell* was the most cited paper in this research area with 135 citations, followed by Heiden MGV’s (Vander Heiden et al., 2009) and Warburg O’s (Warburg, 1956) articles in *Science*, both of which offered crucial insights into tumor metabolism and are landmark events in the metabolism reprogramming of HCC (Figure 6C). In these cited references, the highest number of documents were from 2016 to 2018, indicating a high level of scholarship in this research area during this period (Figure 6D).

**TABLE 2:** Most local cited papers.

Document	DOI	Year	Local citations	Global citations	LC/GC ratio (%)	Normalized local citations	Normalized global citations
Ally A, 2017, Cell	10.1016/j.cell.2017.05.046	2017	22	1,178	1.87	8.48	13.63
Gao Q, 2019, Cell	10.1016/j.cell.2019.08.052	2019	20	404	4.95	7.59	8.69
Senni N, 2019, Gut	10.1136/gutjnl-2017-315448	2019	19	77	24.68	7.21	1.66
Guo WJ, 2015, Hepatology	10.1002/hep.27929	2015	16	180	8.89	4.00	2.07
Shang RZ, 2016, World J Gastroenterology	10.3748/wjg.v22.i45.9933	2016	16	74	21.62	5.82	1.62
Nakagawa H, 2018, Cancers	10.3390/cancers10110447	2018	16	84	19.05	5.91	1.74
Chen CL, 2016, Cell Metab	10.1016/j.cmet.2015.12.004	2016	15	249	6.02	5.45	5.46
Fujiwara N, 2018, Gut	10.1136/gutjnl-2017-315193	2018	14	100	14.00	5.17	2.07
Li JB, 2015, J Hepatology	10.1016/j.jhep.2015.07.039	2015	12	140	8.57	3.00	1.61
Liu MX, 2018, Oncogene	10.1038/s41388-017-0070-6	2018	12	102	11.76	4.43	2.11

### 3.6 Analysis of keywords and trend topics

**FIGURE 7:**
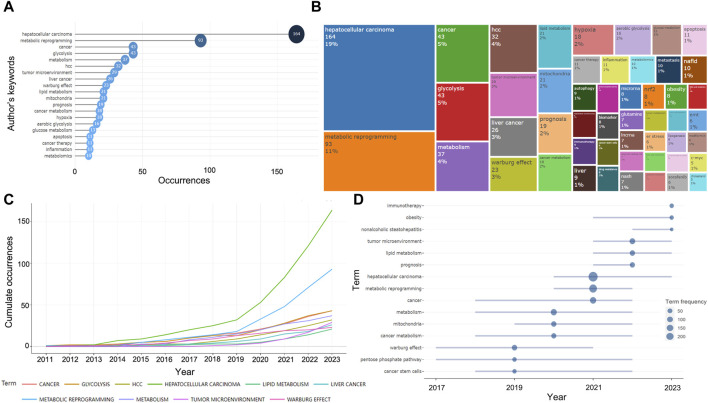
Information about keywords and trend topics. **(A)** Top 20 most frequent keywords in 575 papers. **(B)** The treemap of the top 50 keywords. **(C)** Cumulate the occurrence of the top 20 most frequent keywords in recent years. **(D)** Trend topics in the last 3 years.

A total of 1,204 keywords were collected in 575 documents, and the high-frequency keywords could help us quickly grasp the research hotspots in this field. As can be seen in the Figures 7A, B, excluding the keywords we searched for, “glycolysis,” “tumor microenvironment,” “Warburg effect,” “lipid metabolism,” “mitochondria,” “hypoxia,” and “inflammation” appeared with high frequency, representing the main content of the current research on metabolic reprogramming in HCC. The frequency of these keywords has increased gradually from year to year, especially significantly after 2019, suggesting that metabolic abnormalities in HCC are attracting more and more widespread attention (Figure 7C). We then speculated on the hot topics in future metabolic reprogramming research in HCC based on the temporal development of high-frequency words, which might still be mainly focused on “tumor microenvironment,” “lipid metabolism,” “prognosis,” “Warburg effect,” and “mitochondria” in the future (Figure 7D).

### 3.7 Analysis of clustering and collaborative network

**FIGURE 8:**
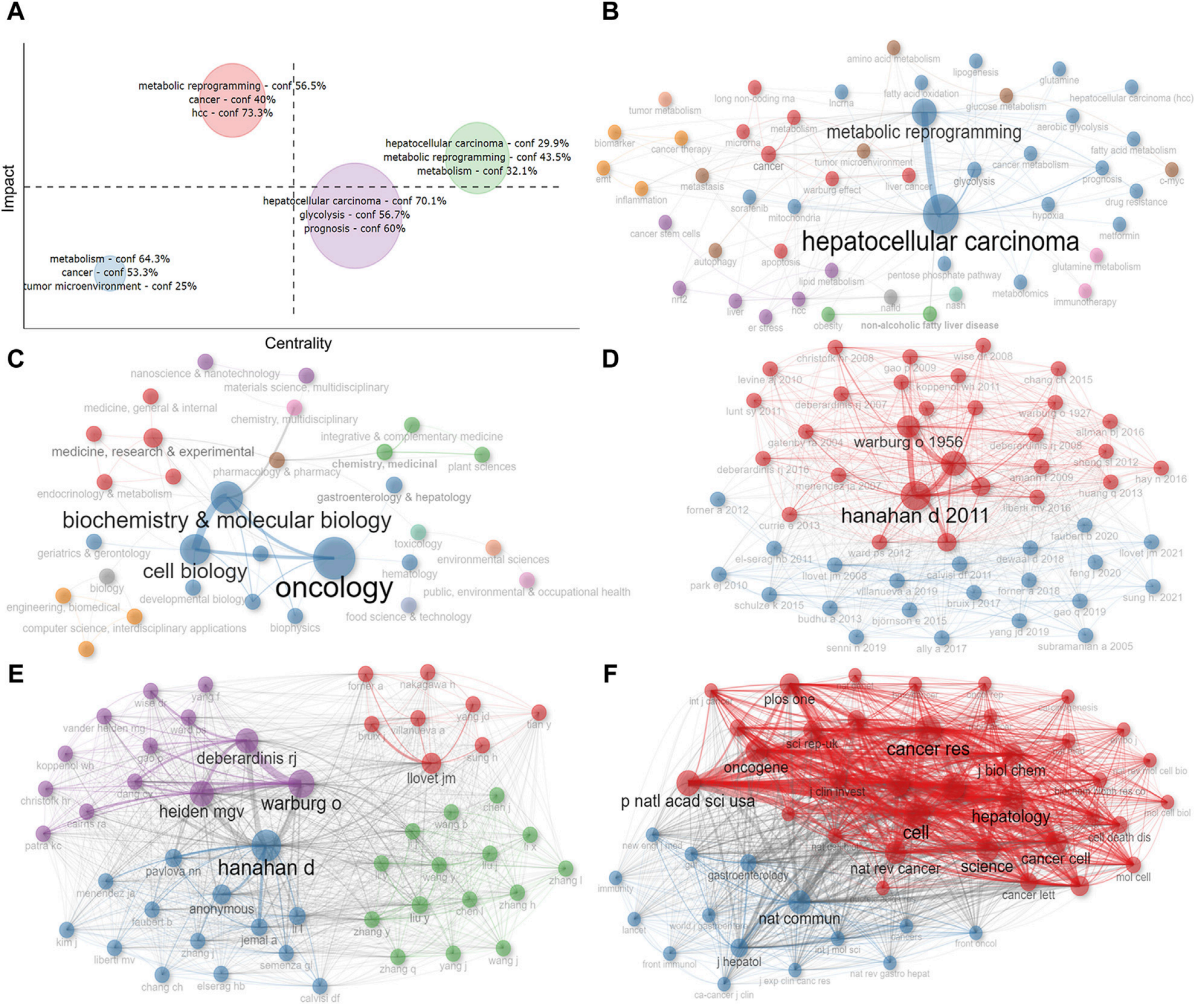
Analysis of cluster and co-citation. **(A)** Cluster by coupling keywords. **(B)** Co-occurrence network of keywords. **(C)** Co-occurrence network of disciplines. **(D)** Co-citation network of papers. **(E)** Co-citation network of authors. **(F)** Co-citation network of publications. Dot size and line thickness were positively correlated with the number of articles and citations, respectively.

To more visually illustrate the linkages between the above key information, we performed clustering and collaboration network analysis. We used the number of global citations as the impact evaluation index and performed cluster analysis based on keywords, and found that there was a total of four major categories involving tumor microenvironment, glycolysis, metabolic reprogramming, and so on, among which glycolysis-related studies were more influential (Figure 8A). Subsequently, we constructed co-occurrence network diagrams between keywords and disciplines, respectively, reflecting the relevance of each topic under this research area (Figures 8B, C). Furthermore, multiple disciplines were also involved, with Oncology, Biochemistry and Molecular Biology, and Cell Biology being the main ones (Figure 8C). Moreover, to discover the co-citation of the 575 studies more visually, we mapped the co-citation network of papers, authors and publications (Figures 8D–F). The results showed that Hanahan D’s paper published in 2011 and the journal *Cell* in which it appeared are at the center of the network.

**FIGURE 9:**
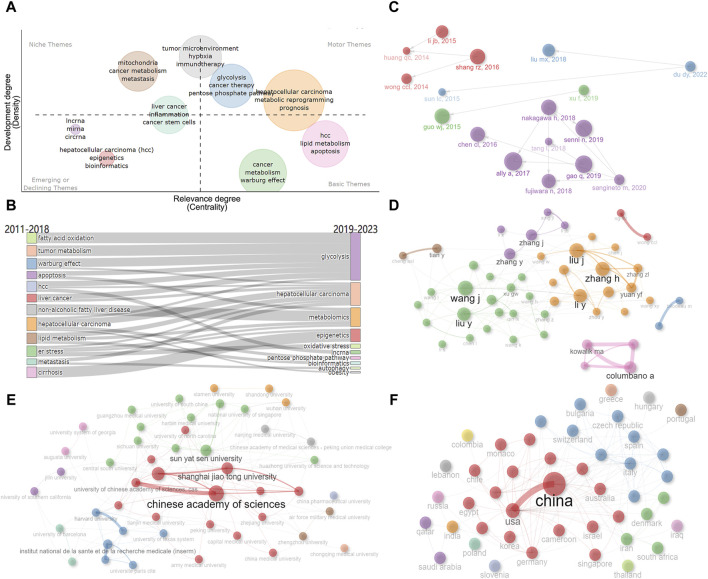
Analysis of themes and social structure. **(A)** The thematic map is created by machine algorithms. **(B)** Evolution of research themes using 2018 as a boundary. **(C)** Historiograph of these papers. **(D)** Author’s collaborative network diagram. **(E)** Institution’s collaborative network diagram. **(F)** Countries’ collaborative network diagram. Dot size and line thickness were positively correlated with the number of articles and the degree of cooperation, respectively.

We further created the thematic map by machine algorithms (Figure 9A), with the themes in the first quadrant (top right) representing themes that are both important and well-developed for this research area and are the focus of current research. The second quadrant (top left) represents topics that have been well-developed but are not as relevant to this research area. The third quadrant (bottom left) represents emerging research themes that are not yet well developed. Quadrant IV (bottom right) generally refers to concepts that are fundamental to this research area and have strong relevance to this research area. As a result, we could see that the impact of metabolic reprogramming on the prognosis of HCC patients, glycolysis, and tumor microenvironment are important themes in this research area. From the number of articles published each year, it can be found that the number of articles increased rapidly after 2018, therefore, we took 2018 as the boundary and speculated the evolution of topics before and after 2018 based on the keywords, and Figure 9B showed that fatty acid oxidation, tumor metabolism, and the Warburg effect all gradually evolved into the study of glycolysis, the study of non-alcoholic fatty liver disease and lipid metabolism gradually evolved into the study of overall metabolomics, the study of endoplasmic reticulum stress gradually evolved into the study of oxidative stress, and so on. Both the Historiograph figure and table show the literature with high importance in this dataset, with Ally et al., (2017) publication having a significant impact on several articles (Figure 9C; Supplementary Table S5).

**FIGURE 10:**
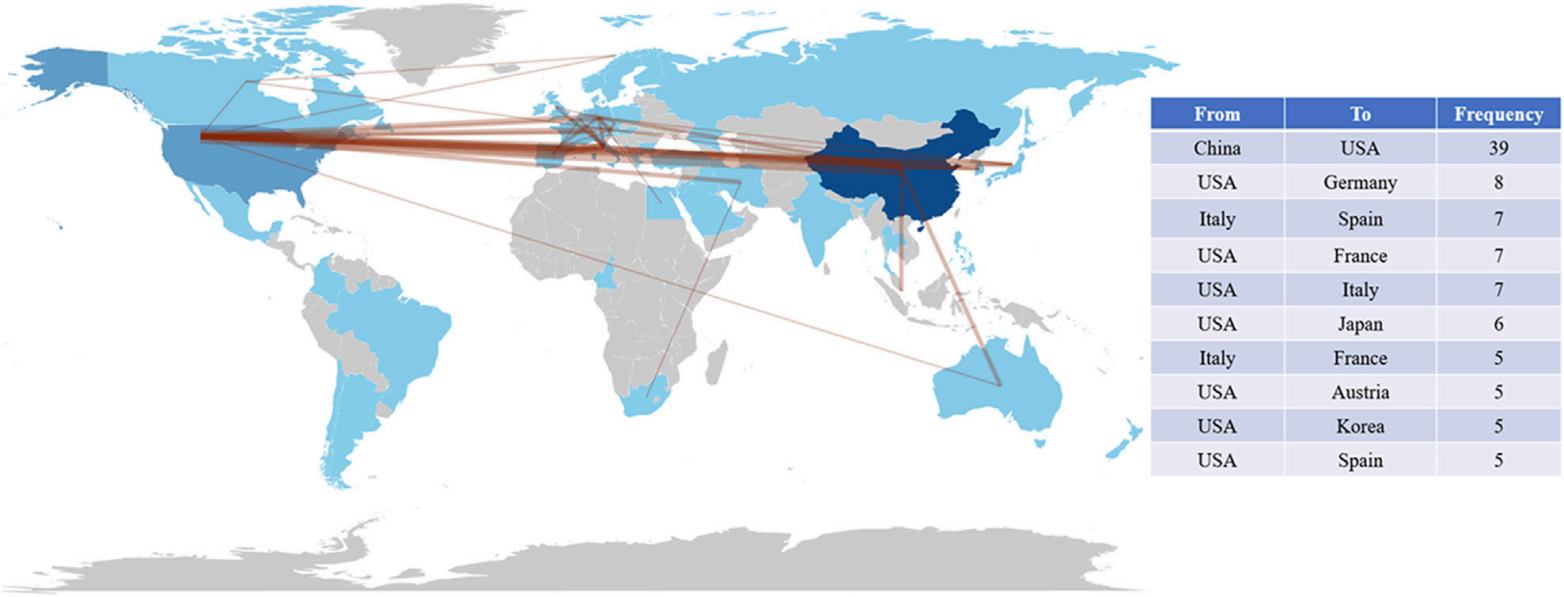
Map of cooperation in countries around the world and the top 10 closely cooperating countries. The thickness of the connecting lines is positively correlated with cooperation between countries.

In addition, we analyzed the collaboration between authors, institutions, and countries (Figures 9D–F). As shown in Figure 9D, there is a closer cooperation between Zhang H and Liu J, Liu Y and Wang J. There is a strong connection between Fudan University, Shanghai Jiaotong University, and the Chinese Academy of Sciences (Figure 9E). The connection between China and the United States is the most, followed by the United States and Germany, Italy and Spain, the United States and France, Italy, Japan, etc. (Figure 9F). It can be seen that the United States maintains close international cooperation with several countries, which plays an important role in the achievement of its high-level results (Figure 10).

## 4 Discussion

### 4.1 General overview

Metabolic reprogramming is a topic that has gained a lot of attention in tumor biology research in recent years. The abnormal changes in the metabolism of glucose, lipids, and amino acids in tumor cells are necessary for their growth, especially in HCC. Studying the mechanisms behind metabolic reprogramming in HCC can help in identifying potential therapeutic targets and developing effective treatment strategies (Ohshima and Morii, 2021). This study used bibliometrics to analyze high-impact publications on metabolic reprogramming in HCC, helping researchers understand the latest trends and research hotspots in this field. The study analyzed 575 openly published articles in WoSCC between 2012 and 2023, identifying key journals, authors, and high-impact papers, as well as analyzing the intellectual structure and social collaborations in this area. This analysis provides valuable insights into the historical development and research frontiers of metabolic reprogramming in HCC.

Our research indicates that there has been a significant increase in the number of publications and total citations on metabolic reprogramming in HCC due to the growing interest in tumor metabolism over the past decade. Researchers have primarily concentrated their work in *Cancers*, *Frontiers in Oncology*, and *International Journal of Molecular Sciences*, which are the core journals in this field. *Cancer Research* and *Hepatology* were the most influential sources of publications based on citation analysis and *International Journal of Molecular Sciences* had the highest H-index. Wang J, Li Y, and Liu J were the most prolific authors, and Saksena G ranked first in terms of citation ranking. In terms of productivity, China and the United States were active countries in this field, with 335 and 69 papers published respectively. Fudan University, Chinese Academy of Sciences, and Sun Yat-sen University were the primary research institutions. Despite having the largest number of publications, China still needs to strengthen its international cooperation. Our study showed that the United States collaborates with most countries, and Iran, Spain, and France have a higher percentage of international collaborations in their total number of publications, suggesting that these countries rely more on international collaborations for research results in this field.

### 4.2 Metabolic reprogramming

Since the Warburg effect has been formally proposed, metabolic abnormalities in tumors have been gradually emphasized, and metabolic reprogramming has been regarded as a major feature of tumors.

#### 4.2.1 Reasons why metabolic reprogramming has emerged

The activities of life are inextricably linked to a corresponding material and energy base, a truth that extends to tumor cells. The unique characteristics of tumor cells require them to proliferate rapidly, evade immune surveillance, and under specific circumstances metastasize to other parts of the body while resisting drug attacks. Consequently, scholars have dedicated significant attention to the energy and material metabolism of tumor cells in search of selective control of certain metabolic pathways to impede the corresponding malignant phenotype. Although the field is still in its nascent stages, there is a history of diagnostic and therapeutic tools developed based on the properties of tumor metabolism. In the field of tumor imaging, for example, the 18F-FDG PET/CT method is frequently employed due to the high glucose uptake capacity of tumors (Cho et al., 2015). Another example is the anticancer drugs 5-fluorouracil and cytosine arabinoside, which are a class of compounds that resist DNA metabolism (De Jager et al., 1976). As research into tumor metabolism advances, we can expect the development of more advanced diagnostic and therapeutic tools.

The study of tumor metabolism is a crucial aspect of cancer diagnosis and treatment. Beyond this, it holds significant implications for daily life. While oncogenic mutations can cause normal cells to become cancerous (Zucman-Rossi et al., 2015), the presence of mutations does not necessarily translate to tumor development. The environment, therefore, plays a critical role in the development of tumors. Consequently, the study of metabolic abnormalities in tumors aims to decipher the influence of environmental factors, such as diet, exercise, and other lifestyle habits, in tumor development, treatment, and prognosis. This knowledge is pivotal for cancer prevention and treatment (Zhang et al., 2021).

Metabolic reprogramming in tumors is a complex phenomenon that is closely related to various factors, including epigenetic regulation, which are classical areas of tumor research, and all of them are inevitably associated with metabolic reprogramming (Xu et al., 2023). Studies have shown that almost all epigenetic modification processes require the participation of metabolites, such as lactate, acetyl coenzyme A, nicotinamide adenine dinucleotide, α-ketoglutarate, succinate, etc., which are used as substrates to participate in post-translational modification processes, such as acetylation, methylation, and phosphorylation (Sun et al., 2022). Thus, metabolites play a very broad and important role in tumor epigenetic modification, drawing the attention of scholars to the metabolic patterns of tumor cells.

#### 4.2.2 The implications of milestone studies for future research

Metabolic studies on liver cancer were first published in 2012. However, the results published by Ally A in 2017 in the journal *Cell* (Ally et al., 2017) are considered a landmark in metabolic reprogramming research in HCC. The study comprehensively analyzed and identified mutated genes in HCC through multi-omics testing of a large number of HCC clinical samples, and identified abnormal genes albumin (ALB), apolipoprotein B (APOB), carbamoyl phosphate synthetase 1 (CPS1) that lead to metabolic reprogramming in HCC. The study suggested that combinations of multiple drugs targeting these abnormal genes can achieve the most effective therapeutic effects (Ally et al., 2017), which provides a clear direction for future research. For example, CPS1 is one of the key enzymes in HCC metabolism, and studies have determined that decreased expression of CPS1 in HCC led to an increase in pyrimidine synthesis, which promoted the development of HCC. The network was filtered to exclude any duplicates, as well as duplicates more than three steps away from CPS1 or carbamoyl phosphate synthetase/aspartate transcarbamoylase/dihydroorotase (CAD). Then CPS1-related metabolites and metabolic gene networks were analyzed by the R package, showing the prognostic role of network genes (Dumenci et al., 2020). In addition to alterations in these specific genes in metabolic reprogramming of HCC, Ally A’s study also identified other important targets such as Wnt signaling, mesenchymal-epithelial transition (Met), vascular endothelial growth factor A (VEGFA), telomerase reverse transcriptase (TERT), and the immune checkpoint proteins CTLA-4, PD-1, and PD-L1, which were regulated by anticancer agents. Researchers have therefore started studying metabolic reprogramming in HCC beyond glycolipid metabolic patterns to better understand its regulatory mechanisms and investigate the causes of reprogramming from upstream pathways. Growing evidence reported the role of Wnt/β-linker signaling in aerobic glycolysis, fatty acid metabolism, and glutamine catabolic synthesis of HCC (Leung and Lee, 2022). Lactate, which is produced by aerobic glycolysis, acidifies the tumor microenvironment and facilitates tumor-associated macrophage (TAM) polarization (San-Millán and Brooks, 2017; de la Cruz-López et al., 2019). Meanwhile, Wnt/β-catenin signaling activated glycolysis and promoted macrophage polarization, leading to endothelial mesenchymal transition (EMT) development and HCC invasion (Jiang et al., 2021). Furthermore, Wnt/β-catenin could regulate glucose metabolism and promote the EMT process by inducing mitochondrial inhibition and glycolytic activation (Lee et al., 2012). The membrane receptor tyrosine kinase Met plays an important role in HCC EMT and mesenchymal phenotype acquisition. Studies have found that hyperglycemia activated Met, which in turn enhanced HCC cell invasion. However, inhibition of Met kinase activity reverses glycolytic gene expression in HCC cells (Topel et al., 2021). Additionally, metformin has been found to reduce phospho-ERK and Cyclin D1 and c-Myc expression in AKT/c-Met mice and hindered the malignant transformation of hepatocytes in an AKT/c-Met-activated HCC mouse model (Zhang et al., 2019). TERT, which synthesizes telomeric DNA to maintain telomere stability, is commonly altered in HCC (Lee et al., 2018). Aerobic glycolysis could also impact tumor biology through epigenetic regulation of tumor-associated genes. Studies have shown that metabolic reprogramming increased the expression of the TERT oncogene through epigenetic changes such as histone acetylation, thereby promoting tumor cell proliferation (Onizuka et al., 2021). In addition, hypoxia-inducible factors 1α (HIF-1α), are often closely associated with tumor progression, and they could promote the acquisition of a malignant phenotype in HCC by activating glycolysis and the transcription of angiogenic cytokines such as VEGF (Hamaguchi et al., 2008; Rigiracciolo et al., 2015). When T cells are activated, they undergo metabolic reprogramming to meet their differentiation and functional expression requirements (Frauwirth and Thompson, 2004). However, studies have confirmed the activated T cells connected to PD-1 shift their metabolic process from glycolysis to enhanced fatty acid oxidation, which activates CPT1A and promotes lipolysis to maintain long-term survival. On the other hand, CTLA-4 inhibited glycolysis through the expression of the glucose transporter protein Glut1, it did not enhance CPT1A and FAO, playing a role in preserving the metabolic profile of unstimulated cells and maintaining immune quiescence (Patsoukis et al., 2015). These findings mechanistically reveal key targets and mechanisms in metabolic reprogramming of HCC, providing intervention targets for subsequent treatment of HCC, and laying a scientific foundation for future research.

#### 4.2.3 The recent studies in the metabolic reprogramming of HCC

A recent study published in *Cell* looks at increased levels of abnormally metabolized arginine in HCC with decreased expression of arginine synthesis genes, suggesting that arginine undergoes metabolic reprogramming with a reduction in arginine-polyamine conversion, ultimately leading to the accumulation of high levels of arginine. Mechanistically, it also discovered that RBM39-mediated upregulation of asparagine synthesis results in enhanced arginine uptake, which helps to maintain high arginine levels and oncogenic metabolism (Mossmann et al., 2023). This study initially focused on the phenotype of abnormal metabolism in HCC and then searched for relevant regulated target genes to elucidate the mechanism of abnormal metabolism in HCC. Similarly, another study found that reduced metabolism of propionyl-CoA due to the downregulation of ALDH6A1 in metabolic reprogramming is closely associated with HCC development. Mechanistically, Pro-CoA produced by ALDH6A1 inhibited the activity of citrate synthase, impaired mitochondrial respiration, and membrane potential, reduced ATP production, and inhibited HCC proliferation (Sun et al., 2023). This study discovered the key pathway to inhibit the growth of HCC by targeting the abnormal metabolic enzymes in HCC cells. Li et al. (2023) identified TK1 as a key driver of metabolic reprogramming in HCC and verified its role in HCC progression by TK1 inhibition and overexpression. Mechanistically, TK1 could bind PRMT1 to promote HCC glycolysis and enhance the malignant phenotype of HCC. All these researches aim to deeply explore the adaptive regulation of key enzymes in HCC metabolic reprogramming, elucidate their molecular features, identify diagnostic and therapeutic targets for HCC, and provide HCC prevention and treatment strategies by intervening in metabolic reprogramming of HCC cells.

### 4.3 Tumor microenvironment

According to current research and predicted future trends in the field, tumor microenvironment (TME) is an important factor that cannot be overlooked. In the TME, interactions between immune cells (the main cell population besides tumor cells) have a significant impact on metabolic reprogramming, which is a key determinant of the antitumor immune response. By understanding the metabolism of immune cells and their interactions with tumor cells, we can come up with new ideas for targeting metabolic pathways in antitumor immunotherapy.

#### 4.3.1 Immune cell metabolism in cancer

Complex metabolic patterns similar to those of tumor cells are also present in immune cells. Under normal circumstances, immune cells maintain only basic nutritional intake, the lowest rate of glycolysis, and biosynthesis level to remain in their resting state. However, when the body is stimulated by external substances like inflammation, these immune cells are activated, resulting in increased energy and biosynthesis demand, leading to significant changes in their metabolic pattern (Pearce et al., 2013).

Activated T cells preferentially use aerobic glycolysis over TCA-coupled OXPHOS for ATP production and biosynthesis, while regulatory T cells (Treg) rely on OXPHOS and FAO to support their survival and differentiation (Beier et al., 2015). Similarly, activated neutrophils (Bodac and Meylan, 2021), M1-type macrophages (Netea-Maier et al., 2018), and dendritic cells (Peng et al., 2021) are primarily dependent on glycolysis for energy. This suggests that different metabolic patterns can influence the differentiation of immune cell subpopulations. However, tumor cells consume most of the nutrients and energy in the TME, hindering the function of immune cells. Therefore, immune cells undergo metabolic reprogramming during proliferation, differentiation, and execution of their functions, which ultimately determines the anti-immune response of the tumor (Leone and Powell, 2020). Macrophages, also known as TAM, are the most critical immune cell population in TME. They can secrete various cytokines and chemokines that promote tumor development. M1 cells perform their pro-inflammatory function mainly through glycolysis, PPP, while M2 cells exert their anti-inflammatory function by enhancing OXPHOS and FAO, however, secretion of IL-1β by M2 cells could lead to proliferation, invasion, and spreading and promote HCC metastasis (Zhang et al., 2018). Neutrophils are one of the major immune cells in TME, also known as tumor-associated neutrophils (TAN). Neutrophil metabolism mainly relies on glycolysis and OXPHOS to produce more ATP (Borregaard and Herlin, 1982; Patel et al., 2018). However, in TME, due to an insufficient amount of glucose, TAN usually utilizes mitochondria for fatty acid oxidation (Patel et al., 2018), which promotes growth and invasion of HCC (Granot and Jablonska, 2015). Tumor-derived hypoxia with lactate accumulation leads to inhibition of glycolysis and upregulation of OXPHOS in dendritic cells. Depletion of extracellular amino acids, lactate accumulation, and nutrient deprivation-induced AMPK activation inhibit TCR signaling and its downstream glycolysis in T effector cells, such as CD8 cytotoxic T cells, while activating OXPHOS and FAO. This leads to the differentiation of Tregs, which promotes immune escape and tumor growth by relying on FAO for energy (Biswas, 2015). Tregs also mainly rely on FAO for energy and play an immunosuppressive role in TME (Michalek et al., 2011). Memory T cells, on the other hand, have a rather unique metabolic state with mitochondrial fatty acid oxidation to maintain energy requirements for basic survival (van der Windt et al., 2012).

#### 4.3.2 Tumor-derived metabolites regulate metabolic reprogramming of immune cells

The TME is an intrinsically self-interested environment that develops during tumor growth. Its purpose is to maintain the rapid proliferation of cancer cells by altering cellular functions and related signaling pathways (Odegaard and Chawla, 2011). The TME and tumor cells have an interaction that is one of the key factors contributing to tumor immune escape (Martínez-Reyes and Chandel, 2021). For example, the accumulation of lactic acid produced by aerobic glycolysis in tumor cells, decreased pH, hypoxia, and enhanced reactive oxygen species (ROS) favor the production of TME, leading to tumor progression and immune escape (Watson et al., 2021). In HCC, succinate levels are elevated (Yang et al., 2023), which promoted the inflammatory state of TME by activating the receptor and amplifying toll-like receptor (TLR) signaling, leading to increased IL-1β secretion (Wu et al., 2020).

TME contains not only cancer cells but also various immune cells. The metabolites produced during tumor metabolism affect the differentiation and metabolic pattern of immune cells. For instance, lactate production through glycolysis in early tumor stages promotes TAM polarization via HIF-1α. Hypoxia in the TME promoted TAM polarization to the M2 phenotype, which activated metabolic reprogramming and facilitated tumor cell proliferation and angiogenesis (Chen X. J. et al., 2019). Excessive lactic acid also inhibited the upregulation of nuclear factor of activated T cell (NFAT) signaling in NK cells, impaired IFN-γ secretion, and promoted apoptosis (Brand et al., 2016). Most tumor cells compete with T cells, NK cells, etc. for arginine uptake, inhibiting their metabolic activity and creating an immunosuppressive microenvironment. However, T cells stimulated by arginine supplementation have significantly enhanced anti-tumor immunity and prolonged the survival time of mice (Davel et al., 2002). Immunosuppressive cells can suppress anti-tumor immunity by degrading arginine, such as TAM M2, tolerogenic DCs, and Treg cells (Buck et al., 2016). T cell activation is extremely sensitive to the concentration of tryptophan in the surrounding environment, and tryptophan is heavily utilized by tumor cells, leading to a deficiency that triggers apoptosis of T cells (Cronin et al., 2019). In addition, fatty acids and cholesterol are energy substances essential for immune cell differentiation and functioning. Abnormal accumulation of short-chain and long-chain fatty acids in immunosuppressive cells is involved in the metabolic reprogramming of these cells in TME (Currie et al., 2013).

#### 4.3.3 Mechanisms regulating metabolic reprogramming of immune cells

Studies have demonstrated that macrophage polarization and metabolism are influenced by multiple signals and pathways, such as HIF, PI3K/AKT, PPAR, and AMPK pathways (Wang et al., 2022). Consequently, modifying the macrophage metabolism pathway can directly impact activated TAM polarization and modulate tumor progression. In addition, it was reported that tumor-associated monocytes in HCC showed enhanced glycolysis, increased expression of the key glycolytic enzyme PFKFB3, and activation of the nuclear factor kappa B signaling pathway mediated the increased expression of PD-L1 (Chen D. P. et al., 2019). Ectosomes PKM2 were also found to promote HCC development by affecting metabolic reprogramming of monocytes in the TME, promoting STAT3 phosphorylation in the nucleus, inducing macrophage differentiation, and secreting the related chemokines CCL1 and CCR8 (Hou et al., 2020). Similarly, T cells in cancer prefer aerobic glycolysis for biosynthesis and nutrient uptake (Fox et al., 2005; Vander Heiden et al., 2009; Chang et al., 2013). However, the metabolic needs of different T cell subsets vary depending on their function. CD4^+^T cells are mainly dependent on glycolysis and fatty acid *de novo* synthesis (Berod et al., 2014), while CD8^+^T cells mainly rely on upregulation of glycolysis, glutamine catabolism, and FAO to exert potent antitumor cytotoxic activity (Pearce et al., 2009), which is associated with the activation of PI3K/Akt/mTOR-related pathway and c-Myc, glycolytic genes (GLUT1, PDK1, or HK2) (MacIver et al., 2013; Maciolek et al., 2014). Studies have shown that IFN-α improved glucose metabolism in the TME by inhibiting HIF-1α signaling, decreasing glucose consumption, activating mTOR-FOXM1 signaling, promoting the toxic effects of CD8^+^T cells, and enhancing PL-D-blocked immune responses to achieve anti-HCC (Hu et al., 2022). Moreover, the mTOR and the AMPK signaling pathway are extensively involved in the metabolic regulation of immune cells. mTORC1 promoted the expression of PD-L1, inhibited the infiltration of NK cells and T cells in the tumor immune microenvironment, and allowed tumor cells to evade killing by immune cells (Mafi et al., 2021). Activation of the AMPK signaling pathway is involved in macrophage polarization as well as T lymphocyte differentiation (Keerthana et al., 2023).

Overall, multiple metabolic pathways of immune cells like glycolysis and FAO in TME are designed for anti-tumor immune responses and pro-tumor immune escape, such as the deletion of M1 macrophages, N1 neutrophils, and CD8^+^T cells, and the activation of M2 macrophages, N2 neutrophils, and Treg cells (Xia Y. J. et al., 2021). Meanwhile, metabolites and cytokines generated during metabolic reprogramming can promote the establishment of TME. For example, accumulation of lactate, low glucose, and a hypoxic state all promote rapid proliferation and metastasis of HCC. Nevertheless, the complex roles and potential mechanisms between the tumor microenvironment and metabolic reprogramming in HCC are not yet fully understood. Further research in this area can enhance our understanding of HCC pathogenesis and inform the development of clinical therapeutic strategies.

### 4.4 Implications and recommendations for future research

Numerous studies have substantiated that HCC cells undergo significant metabolic reprogramming when compared to normal hepatocytes. It is mainly manifested in the abnormally active glycolysis, fatty acid synthesis, and glutamine metabolism, which is related to the overexpression and activation of the key enzymes or pathways that regulate these processes. Several investigations have demonstrated that targeting these abnormal metabolic enzymes and pathways in HCC cells can significantly impede HCC growth and metastasis. As such, this presents a promising avenue for clinical application in the management of HCC (Foglia et al., 2023). However, there are still some issues with the diagnosis and treatment of tumors marked by metabolic enzymes and pathways that deserve attention and in-depth research. Besides, these metabolites and key enzymes have not been quantified yet. On the one hand, individual metabolic enzymes or products tend to be less effective as diagnostic markers, whereas combining multiple metabolic markers shows better results. On the other hand, most studies are centered around one or a few metabolic enzymes or pathways, which are relatively inefficient for research purposes. Due to the existence of complex metabolic compensation mechanisms in tumor cells, targeting a single metabolic enzyme or pathway often has a very limited inhibitory effect on tumors. The combination of multiple metabolic targets may enhance the killing effect on tumors, which is the most attractive direction for tumor targeted therapy in the future. With the development of proteomics and metabolomics, the joint multi-omics analysis will facilitate researchers to conduct a comprehensive and systematic study of the metabolic reprogramming network of HCC cells more efficiently, which will assist in the search for new and more critical metabolic enzymes and metabolites of HCC abnormalities. Identification of key enzymes involved in metabolic reprogramming in HCC by visualization and quantification techniques could be a valuable reference for targeting HCC therapy.

There may be a synergistic relationship between metabolic reprogramming and the TME. A deeper understanding and appropriate utilization of the cross-talk between the two has the potential to enhance the efficacy of tumor immunotherapy and ameliorate the low response rate to immunotherapy. Moreover, immune metabolism in tumors presents a new research field, and interfering with the metabolic reprogramming of immune cells can not only affect the function of HCC cells but also change the immunosuppression in the TME, which provides a promising therapeutic strategy for the future (Xia L. et al., 2021). In recent years, the fields of tumor metabolism, immune metabolism, and immunotherapy have experienced significant progress, but studies exploring the association between these fields have been insufficient. The appropriate utilization of these strategies in the complex tumor microenvironment remains uncertain. Although metabolic drugs in combination with immunotherapy have been used in clinical trials, they are still in their infancy. To optimize the combination strategies of metabolic and immune drugs, it is imperative to gain a deeper understanding of the relationship between metabolic reprogramming and immune cells in HCC. This necessitates restoring physiological conditions from clinical to animal experiments, from cellular to molecular levels, clarifying targets, and elucidating mechanisms. However, this process requires extensive clinical trials and animal model validation, which can be costly in terms of time and economics. Nevertheless, the emergence of new biological technologies, such as organoids, stem cells, microfluidics, and nano-drug delivery systems, has ushered in a new era of research. These emerging technologies can provide crucial evidence for studying tumor metabolism and immune interactions, and they represent a key focus for future development.

This scientific study analyzed the metabolic reprogramming in HCC from 2012 to 2023 using the R package software tool. The study used scientific and rigorous methods to evaluate and screen the literature, which enabled a more comprehensive collection of relevant information on metabolic reprogramming in HCC than traditional narrative reviews. The study analyzed the most representative articles, authors, journals, and countries in the field, and also analyzed the co-citations and collaborations among them. Notably, the study visually demonstrated the research development history, current research status, research hotspots, and developmental trends, which provided valuable insights for researchers. According to the study, metabolic reprogramming in HCC is undergoing a rapid development stage, with a primary focus on the regulation of metabolic enzymes and key pathways behind the abnormal glucose, lipid, and glutamine metabolism in HCC cells. This development is linked to the TME immune cell metabolism, which has become a new research hotspot and trend in this field. The study’s findings indicate that metabolic reprogramming in HCC is a dynamic and rapidly evolving field with significant potential for future research. It is worth noting that as a systematic review, meta-analysis may not be able to summarize and organize the latest research progress in this field and provide academic insights well, as the field mostly focuses on basic research. Therefore, it is essential to utilize other methods to complement and supplement the findings of analytical studies. Overall, this study’s findings provide a valuable overview of the research progress in metabolic reprogramming in HCC, which will be useful for researchers in this field.

### 4.5 Limitations and strengths

This study comprehensively analyzes the research characteristics and trends of metabolic reprogramming in HCC, which will provide new insights into the diagnosis, prognosis, and identification of therapeutic targets for HCC. However, there are some technical limitations to this study.

Although WoSCC is a highly regarded academic database worldwide, its coverage remains limited, particularly in non-English-speaking countries and regions. Consequently, the study’s inclusion of literature from WoSCC may not be comprehensive enough. However, it is essential to note that the WoSCC database provides researchers with high-quality literature content that is thoroughly screened and vetted to ensure credibility and accuracy. It also offers reliable citations of the literature and comprehensive assessments of scholarly achievements. Therefore, despite the fact that only WoSCC literature was included in this study, the results remained largely unaffected. Nevertheless, to ensure a comprehensive assessment and avoid potential impacts, it is advisable to consider the inclusion of several more databases in future studies.

In addition, the statistical analysis of literature is often marred by issues of sample selection bias and endogeneity. The use of the number of citations and H-index to measure the impact of papers is a prime example of such biases. The metrics employed are limited to the output and impact of published papers, ignoring their quality. Moreover, the method does not account for the impact of different stages of the life cycle in which a discipline or field is found. Therefore, to obtain the most representative results in the research field, more paper evaluation indices need inclusion. The choice of measurement methods should depend on the purpose of the study and the characteristics of the data to select appropriate statistical models and analysis methods. The interpretation of the resulting measurements should also account for the inevitable selection bias, thereby ensuring the development of an objective and fair statistical report.

In the context of predicting thematic trends within a research field, it is important to note that the frequency of first-level subject terms may not serve as an accurate indicator for analyzing the specific research direction of the field. Instead, a more comprehensive approach involves extracting the second-level subject terms for analysis. While co-occurrence analysis and citation network analysis can reveal the structure and development of the research field, these methods may not adequately capture rapid changes in emerging fields or trends in non-mainstream research. In addition, predicting future research hotspots and directions can be uncertain in the face of disruptive technologies and swift changes within a specific field. To enhance the accuracy and depth of analysis, methods and techniques from other disciplines like artificial intelligence and machine learning can be applied in the future. Researchers are actively developing new predictive models and methods to improve their ability to anticipate future research trends.

Finally, it should be noted that despite the strong functionality of the biblioshiny tool we employed, it is not without its limitations. Thus, it is recommended that it be complemented with other literature analysis software to enhance its strengths in the future. A promising area of development that can be utilized for this purpose is Webometrics, which incorporates traditional literature bibliometric methods on web pages. This is particularly valuable in the context of the current network information age, providing comprehensive statistics on the development trends in a particular research direction, which has significant potential for broad applications.

## 5 Conclusion

In summary, this study utilized bibliometrix and visual analysis to provide a comprehensive summary of the most significant research papers concerning HCC metabolism in the extant literature. This analysis effectively identified crucial contributions that have advanced the discipline and the field over the past decade. In particular, the trend in HCC metabolic research is moving away from isolated studies of glucose and lipid metabolism and towards more comprehensive, holistic investigations of metabolic reprogramming. Moreover, these studies are progressively taking into account the increasingly close relationship between the TME and metabolic reprogramming, making the combination of targeted metabolism and immunotherapy a possible new strategy for HCC control in the future, thus providing new insights and research directions for the field.

## Figures and Tables

**Table:** 

**HCC**	Hepatocellular carcinoma
**WoSCC**	Web of Science Core Collection
**NAFLD**	Non-alcoholic fatty liver disease
**MAFLD**	Metabolic dysfunction-associated fatty liver disease
**OXPHOS**	Oxidative phosphorylation
**PPP**	Pentose phosphate pathway
**TCA**	Tricarboxylic acid
**FAO**	Fatty acid β-oxidation
**SCIE**	Science Citation Index Expanded
**IF**	Impact factor
**TC**	Total citation
**ALB**	Albumin
**APOB**	Apolipoprotein B
**CPS1**	Carbamoyl phosphate synthetase 1
**CAD**	Carbamoyl phosphate synthetase/aspartate transcarbamoylase/dihydroorotase
**Met**	Mesenchymal-epithelial transition
**VEGFA**	Vascular endothelial growth factor A
**NFAT**	Nuclear factor of activated T cell
**TAM**	Tumor-associated macrophage
**EMT**	Endothelial mesenchymal transition
**TERT**	Telomerase reverse transcriptase
**TLR**	Toll-like receptor
**TME**	Tumor microenvironment
**ROS**	Reactive oxygen species
**TAN**	Tumor-associated neutrophils

## Data Availability

The original contributions presented in the study are included in the article/Supplementary Material, further inquiries can be directed to the corresponding author.

## References

[B1] Ally A. Balasundaram M. Carlsen R. Chuah E. Clarke A. Dhalla N. (2017). Comprehensive and integrative genomic characterization of hepatocellular carcinoma. Cell 169, 1327–1341.e23. 10.1016/j.cell.2017.05.046 28622513 PMC5680778

[B2] Amann T. Maegdefrau U. Hartmann A. Agaimy A. Marienhagen J. Weiss T. S. (2009). GLUT1 expression is increased in hepatocellular carcinoma and promotes tumorigenesis. Am. J. Pathol. 174, 1544–1552. 10.2353/ajpath.2009.080596 19286567 PMC2671384

[B3] Anstee Q. M. Reeves H. L. Kotsiliti E. Govaere O. Heikenwalder M. (2019). From NASH to HCC: current concepts and future challenges. Nat. Rev. Gastroenterol. Hepatol. 16, 411–428. 10.1038/s41575-019-0145-7 31028350

[B4] Aria M. Cuccurullo C. (2017). Bibliometrix: an R-tool for comprehensive science mapping analysis. J. Informetr. 11, 959–975. 10.1016/j.joi.2017.08.007

[B5] Beier U. H. Angelin A. Akimova T. Wang L. Liu Y. Xiao H. (2015). Essential role of mitochondrial energy metabolism in Foxp3⁺ T-regulatory cell function and allograft survival. FASEB J. 29, 2315–2326. 10.1096/fj.14-268409 25681462 PMC4447222

[B6] Berod L. Friedrich C. Nandan A. Freitag J. Hagemann S. Harmrolfs K. (2014). *De novo* fatty acid synthesis controls the fate between regulatory T and T helper 17 cells. Nat. Med. 20, 1327–1333. 10.1038/nm.3704 25282359

[B7] Biswas S. K. (2015). Metabolic reprogramming of immune cells in cancer progression. Immunity 43, 435–449. 10.1016/j.immuni.2015.09.001 26377897

[B8] Bodac A. Meylan E. (2021). Neutrophil metabolism in the cancer context. Semin. Immunol. 57, 101583. 10.1016/j.smim.2021.101583 34963565

[B9] Borregaard N. Herlin T. (1982). Energy metabolism of human neutrophils during phagocytosis. J. Clin. Invest. 70, 550–557. 10.1172/jci110647 7107894 PMC370256

[B10] Brand A. Singer K. Koehl G. E. Kolitzus M. Schoenhammer G. Thiel A. (2016). LDHA-associated lactic acid production blunts tumor immunosurveillance by T and NK cells. Cell Metab. 24, 657–671. 10.1016/j.cmet.2016.08.011 27641098

[B11] Buck M. D. O'sullivan D. Klein Geltink R. I. Curtis J. D. Chang C. H. Sanin D. E. (2016). Mitochondrial dynamics controls T cell fate through metabolic programming. Cell 166, 63–76. 10.1016/j.cell.2016.05.035 27293185 PMC4974356

[B12] Chai F. Li Y. Liu K. Y. Li Q. Sun H. Z. (2019). Caveolin enhances hepatocellular carcinoma cell metabolism, migration, and invasion *in vitro* via a hexokinase 2-dependent mechanism. J. Cell. Physiol. 234, 1937–1946. 10.1002/jcp.27074 30144070

[B13] Chang C. H. Curtis J. D. Maggi L. B. Faubert B. Villarino A. V. O'sullivan D. (2013). Posttranscriptional control of T cell effector function by aerobic glycolysis. Cell 153, 1239–1251. 10.1016/j.cell.2013.05.016 23746840 PMC3804311

[B14] Chen D. P. Ning W. R. Jiang Z. Z. Peng Z. P. Zhu L. Y. Zhuang S. M. (2019a). Glycolytic activation of peritumoral monocytes fosters immune privilege via the PFKFB3-PD-L1 axis in human hepatocellular carcinoma. J. Hepatol. 71, 333–343. 10.1016/j.jhep.2019.04.007 31071366

[B15] Chen X. J. Wu S. Yan R. M. Fan L. S. Yu L. Zhang Y. M. (2019b). The role of the hypoxia-Nrp-1 axis in the activation of M2-like tumor-associated macrophages in the tumor microenvironment of cervical cancer. Mol. Carcinog. 58, 388–397. 10.1002/mc.22936 30362630

[B16] Cho E. Jun C. H. Kim B. S. Son D. J. Choi W. S. Choi S. K. (2015). 18F-FDG PET CT as a prognostic factor in hepatocellular carcinoma. Turk. J. Gastroenterol. 26, 344–350. 10.5152/tjg.2015.0152 26039005

[B17] Crane H. Gofton C. Sharma A. George J. (2023). MAFLD: an optimal framework for understanding liver cancer phenotypes. J. Gastroenterol. 58, 947–964. 10.1007/s00535-023-02021-7 37470858 PMC10522746

[B18] Cronin S. J. F. Seehus C. Weidinger A. Talbot S. Reissig S. Seifert M. (2019). Publisher Correction: the metabolite BH4 controls T cell proliferation in autoimmunity and cancer. Nature 572, E18. 10.1038/s41586-019-1459-x 31363232

[B19] Currie E. Schulze A. Zechner R. Walther T. C. Farese R. V. Jr. (2013). Cellular fatty acid metabolism and cancer. Cell. Metab. 18, 153–161. 10.1016/j.cmet.2013.05.017 23791484 PMC3742569

[B20] Davel L. E. Jasnis M. A. De La Torre E. Gotoh T. Diament M. Magenta G. (2002). Arginine metabolic pathways involved in the modulation of tumor-induced angiogenesis by macrophages. FEBS Lett. 532, 216–220. 10.1016/s0014-5793(02)03682-7 12459493

[B21] De Jager R. L. Magill G. B. Golbey R. B. Krakoff I. H. (1976). Combination chemotherapy with mitomycin C, 5-fluorouracil, and cytosine arabinoside in gastrointestinal cancer. Cancer Treat. Rep. 60, 1373–1375. 1016970

[B22] De La Cruz-López K. G. Castro-Muñoz L. J. Reyes-Hernández D. O. García-Carrancá A. Manzo-Merino J. (2019). Lactate in the regulation of tumor microenvironment and therapeutic approaches. Front. Oncol. 9, 1143. 10.3389/fonc.2019.01143 31737570 PMC6839026

[B23] Dewaal D. Nogueira V. Terry A. R. Patra K. C. Jeon S. M. Guzman G. (2018). Hexokinase-2 depletion inhibits glycolysis and induces oxidative phosphorylation in hepatocellular carcinoma and sensitizes to metformin. Nat. Commun. 9, 446. 10.1038/s41467-017-02733-4 29386513 PMC5792493

[B24] Du D. Liu C. Qin M. Zhang X. Xi T. Yuan S. (2022). Metabolic dysregulation and emerging therapeutical targets for hepatocellular carcinoma. Acta Pharm. Sin. B 12, 558–580. 10.1016/j.apsb.2021.09.019 35256934 PMC8897153

[B25] Dumenci O. E. Abellona M. R. U. Khan S. A. Holmes E. Taylor-Robinson S. D. (2020). <p>Exploring Metabolic Consequences of<em> CPS1</em> and<em> CAD</em> Dysregulation in Hepatocellular Carcinoma by Network Reconstruction</p&gt;. J. Hepatocell. Carcinoma 7, 1–9. 10.2147/JHC.S239039 32021853 PMC6955626

[B26] Eslam M. Sanyal A. J. George J. International Consensus P. (2020). MAFLD: a consensus-driven proposed nomenclature for metabolic associated fatty liver disease. Gastroenterology 158, 1999–2014.e1. 10.1053/j.gastro.2019.11.312 32044314

[B27] Estes C. Razavi H. Loomba R. Younossi Z. Sanyal A. J. (2018). Modeling the epidemic of nonalcoholic fatty liver disease demonstrates an exponential increase in burden of disease. Hepatology 67, 123–133. 10.1002/hep.29466 28802062 PMC5767767

[B28] Foglia B. Beltra M. Sutti S. Cannito S. (2023). Metabolic reprogramming of HCC: a new microenvironment for immune responses. Int. J. Mol. Sci. 24, 7463. 10.3390/ijms24087463 37108625 PMC10138633

[B29] Fox C. J. Hammerman P. S. Thompson C. B. (2005). Fuel feeds function: energy metabolism and the T-cell response. Nat. Rev. Immunol. 5, 844–852. 10.1038/nri1710 16239903

[B30] Frauwirth K. A. Thompson C. B. (2004). Regulation of T lymphocyte metabolism. J. Immunol. 172, 4661–4665. 10.4049/jimmunol.172.8.4661 15067038

[B31] Granot Z. Jablonska J. (2015). Distinct functions of neutrophil in cancer and its regulation. Mediat. Inflamm. 2015, 701067. 10.1155/2015/701067 PMC4663337 26648665

[B32] Hamaguchi T. Iizuka N. Tsunedomi R. Hamamoto Y. Miyamoto T. Iida M. (2008). Glycolysis module activated by hypoxia-inducible factor 1alpha is related to the aggressive phenotype of hepatocellular carcinoma. Int. J. Oncol. 33, 725–731. 10.3892/ijo_00000058 18813785

[B33] Hanahan D. Weinberg R. A. (2011). Hallmarks of cancer: the next generation. Cell 144, 646–674. 10.1016/j.cell.2011.02.013 21376230

[B34] Hassan W. Zafar M. Duarte A. E. Kamdem J. P. Da Rocha J. B. T. (2021). Pharmacological Research: a bibliometric analysis from 1989 to 2019. Pharmacol. Res. 169, 105645. 10.1016/j.phrs.2021.105645 33957268

[B35] Hou P. P. Luo L. J. Chen H. Z. Chen Q. T. Bian X. L. Wu S. F. (2020). Ectosomal PKM2 promotes HCC by inducing macrophage differentiation and remodeling the tumor microenvironment. Mol. Cell 78, 1192–1206. 10.1016/j.molcel.2020.05.004 32470318

[B36] Hu B. Yu M. Ma X. Sun J. Liu C. Wang C. (2022). IFNα potentiates anti-PD-1 efficacy by remodeling glucose metabolism in the hepatocellular carcinoma microenvironment. Cancer Discov. 12, 1718–1741. 10.1158/2159-8290.CD-21-1022 35412588

[B37] Huang D. Q. El-Serag H. B. Loomba R. (2021). Global epidemiology of NAFLD-related HCC: trends, predictions, risk factors and prevention. Nat. Rev. Gastroenterol. Hepatol. 18, 223–238. 10.1038/s41575-020-00381-6 33349658 PMC8016738

[B38] Huang Q. Tan Y. X. Yin P. Y. Ye G. Z. Gao P. Lu X. (2013). Metabolic characterization of hepatocellular carcinoma using nontargeted tissue metabolomics. Cancer Res. 73, 4992–5002. 10.1158/0008-5472.CAN-13-0308 23824744

[B39] Iarc W. H. O. (2020). GLOBAL CANCER OBSERVATORY. Available at: https://gco.iarc.fr/(Accessed November 22, 2023).

[B40] Jiang Y. Han Q. J. Zhao H. J. Zhang J. (2021). Promotion of epithelial-mesenchymal transformation by hepatocellular carcinoma-educated macrophages through Wnt2b/β-catenin/c-Myc signaling and reprogramming glycolysis. J. Exp. Clin. Cancer Res. 40, 13. 10.1186/s13046-020-01808-3 33407720 PMC7788901

[B41] Keerthana C. K. Rayginia T. P. Shifana S. C. Anto N. P. Kalimuthu K. Isakov N. (2023). The role of AMPK in cancer metabolism and its impact on the immunomodulation of the tumor microenvironment. Front. Immunol. 14, 1114582. 10.3389/fimmu.2023.1114582 36875093 PMC9975160

[B42] Khan A. S. Rehman S. U. Ahmad S. Almaimouni Y. K. Alzamil M. a.S. Dummer P. M. H. (2021). Five decades of the international endodontic journal: bibliometric overview 1967-2020. Int. Endod. J. 54, 1819–1839. 10.1111/iej.13595 34196006

[B43] Lee M. Ko H. Yun M. J. (2018). Cancer metabolism as a mechanism of treatment resistance and potential therapeutic target in hepatocellular carcinoma. Yonsei Med. J. 59, 1143–1149. 10.3349/ymj.2018.59.10.1143 30450847 PMC6240564

[B44] Lee S. Y. Jeon H. M. Ju M. K. Kim C. H. Yoon G. Han S. I. (2012). Wnt/Snail signaling regulates cytochrome C oxidase and glucose metabolism. Cancer Res. 72, 3607–3617. 10.1158/0008-5472.CAN-12-0006 22637725

[B45] Leone R. D. Powell J. D. (2020). Metabolism of immune cells in cancer. Nat. Rev. Cancer 20, 516–531. 10.1038/s41568-020-0273-y 32632251 PMC8041116

[B46] Leung R. W. H. Lee T. K. W. (2022). Wnt/β-Catenin signaling as a driver of stemness and metabolic reprogramming in hepatocellular carcinoma. Cancers 14, 5468. 10.3390/cancers14215468 36358885 PMC9656505

[B47] Li Q. Zhang L. Yang Q. Li M. Pan X. Xu J. (2023). Thymidine kinase 1 drives hepatocellular carcinoma in enzyme-dependent and -independent manners. Cell Metab. 35, 912–927.e7. 10.1016/j.cmet.2023.03.017 37071992

[B48] Llovet J. M. Kelley R. K. Villanueva A. Singal A. G. Pikarsky E. Roayaie S. (2021). Hepatocellular carcinoma. Nat. Rev. Dis. Prim. 7, 6. 10.1038/s41572-020-00240-3 33479224 PMC13537433

[B49] Llovet J. M. Zucman-Rossi J. Pikarsky E. Sangro B. Schwartz M. Sherman M. (2016). Hepatocellular carcinoma. Nat. Rev. Dis. Prim. 2, 16018. 10.1038/nrdp.2016.18 27158749

[B50] Lu M. Lu L. Dong Q. Z. Yu G. Y. Chen J. H. Qin L. X. (2018). Elevated G6PD expression contributes to migration and invasion of hepatocellular carcinoma cells by inducing epithelial-mesenchymal transition. Acta Biochim. Biophys. Sin. 50, 370–380. 10.1093/abbs/gmy009 29471502

[B51] Maciolek J. A. Pasternak J. A. Wilson H. L. (2014). Metabolism of activated T lymphocytes. Curr. Opin. Immunol. 27, 60–74. 10.1016/j.coi.2014.01.006 24556090

[B52] Maciver N. J. Michalek R. D. Rathmell J. C. (2013). Metabolic regulation of T lymphocytes. Annu. Rev. Immunol. 31, 259–283. 10.1146/annurev-immunol-032712-095956 23298210 PMC3606674

[B53] Mafi S. Mansoori B. Taeb S. Sadeghi H. Abbasi R. Cho W. C. (2021). mTOR-mediated regulation of immune responses in cancer and tumor microenvironment. Front. Immunol. 12, 774103. 10.3389/fimmu.2021.774103 35250965 PMC8894239

[B54] Martínez-Reyes I. Chandel N. S. (2021). Cancer metabolism: looking forward. Nat. Rev. Cancer 21, 669–680. 10.1038/s41568-021-00378-6 34272515

[B55] Michalek R. D. Gerriets V. A. Jacobs S. R. Macintyre A. N. Maciver N. J. Mason E. F. (2011). Cutting edge: distinct glycolytic and lipid oxidative metabolic programs are essential for effector and regulatory CD4+ T cell subsets. J. Immunol. 186, 3299–3303. 10.4049/jimmunol.1003613 21317389 PMC3198034

[B56] Mossmann D. Muller C. Park S. Ryback B. Colombi M. Ritter N. (2023). Arginine reprograms metabolism in liver cancer via RBM39. Cell 186, 5068–5083.e23. 10.1016/j.cell.2023.09.011 37804830 PMC10642370

[B57] Netea-Maier R. T. Smit J. W. A. Netea M. G. (2018). Metabolic changes in tumor cells and tumor-associated macrophages: a mutual relationship. Cancer Lett. 413, 102–109. 10.1016/j.canlet.2017.10.037 29111350

[B58] Ninkov A. Frank J. R. Maggio L. A. (2022). Bibliometrics: methods for studying academic publishing. Perspect. Med. Educ. 11, 173–176. 10.1007/s40037-021-00695-4 34914027 PMC9240160

[B59] Nwosu Z. C. Megger D. A. Hammad S. Sitek B. Roessler S. Ebert M. P. (2017). Identification of the consistently altered metabolic targets in human hepatocellular carcinoma. Cell. Mol. Gastroenterol. Hepatol. 4, 303–323. 10.1016/j.jcmgh.2017.05.004 28840186 PMC5560912

[B60] Odegaard J. I. Chawla A. (2011). Alternative macrophage activation and metabolism. Annu. Rev. Pathol. 6, 275–297. 10.1146/annurev-pathol-011110-130138 21034223 PMC3381938

[B61] Ohshima K. Morii E. (2021). Metabolic reprogramming of cancer cells during tumor progression and metastasis. Metabolites 11, 28. 10.3390/metabo11010028 33401771 PMC7824065

[B62] Onizuka H. Masui K. Amano K. Kawamata T. Yamamoto T. Nagashima Y. (2021). Metabolic reprogramming drives pituitary tumor growth through epigenetic regulation of TERT. Acta histochem. cytochem. 54, 87–96. 10.1267/ahc.21-00007 34276102 PMC8275863

[B63] Patel S. Fu S. Mastio J. Dominguez G. A. Purohit A. Kossenkov A. (2018). Unique pattern of neutrophil migration and function during tumor progression. Nat. Immunol. 19, 1236–1247. 10.1038/s41590-018-0229-5 30323345 PMC6195445

[B64] Patsoukis N. Bardhan K. Chatterjee P. Sari D. Liu B. L. Bell L. N. (2015). PD-1 alters T-cell metabolic reprogramming by inhibiting glycolysis and promoting lipolysis and fatty acid oxidation. Nat. Commun. 6, 6692. 10.1038/ncomms7692 25809635 PMC4389235

[B65] Pearce E. L. Poffenberger M. C. Chang C. H. Jones R. G. (2013). Fueling immunity: insights into metabolism and lymphocyte function. Science 342, 1242454. 10.1126/science.1242454 24115444 PMC4486656

[B66] Pearce E. L. Walsh M. C. Cejas P. J. Harms G. M. Shen H. Wang L. S. (2009). Enhancing CD8 T‐cell memory by modulating fatty acid metabolism. Nature 460, 103–107. 10.1038/nature08097 19494812 PMC2803086

[B67] Peng X. He Y. Huang J. Tao Y. Liu S. (2021). Metabolism of dendritic cells in tumor microenvironment: for immunotherapy. Front. Immunol. 12, 613492. 10.3389/fimmu.2021.613492 33732237 PMC7959811

[B68] Rigiracciolo D. C. Scarpelli A. Lappano R. Pisano A. Santolla M. F. De Marco P. (2015). Copper activates HIF-1α/GPER/VEGF signalling in cancer cells. Oncotarget 6, 34158–34177. 10.18632/oncotarget.5779 26415222 PMC4741443

[B69] San-Millán I. Brooks G. A. (2017). Reexamining cancer metabolism: lactate production for carcinogenesis could be the purpose and explanation of the Warburg Effect. Carcinogenesis 38, 119–133. 10.1093/carcin/bgw127 27993896 PMC5862360

[B70] Schwartzenberg-Bar-Yoseph F. Armoni M. Karnieli E. (2004). The tumor suppressor p53 down-regulates glucose transporters GLUT1 and GLUT4 gene expression. Cancer Res. 64, 2627–2633. 10.1158/0008-5472.can-03-0846 15059920

[B71] Senni N. Savall M. Granados D. C. Alves-Guerra M. C. Sartor C. Lagoutte I. (2019). β-catenin-activated hepatocellular carcinomas are addicted to fatty acids. Gut 68, 322–334. 10.1136/gutjnl-2017-315448 29650531

[B72] Sun J. Ding J. Shen Q. Wang X. Wang M. Huang Y. (2023). Decreased propionyl-CoA metabolism facilitates metabolic reprogramming and promotes hepatocellular carcinoma. J. Hepatol. 78, 627–642. 10.1016/j.jhep.2022.11.017 36462680

[B73] Sun L. Zhang H. Gao P. (2022). Metabolic reprogramming and epigenetic modifications on the path to cancer. Protein Cell 13, 877–919. 10.1007/s13238-021-00846-7 34050894 PMC9243210

[B74] Tan D. J. H. Ng C. H. Lin S. Y. Pan X. H. Tay P. Lim W. H. (2022). Clinical characteristics, surveillance, treatment allocation, and outcomes of non-alcoholic fatty liver disease-related hepatocellular carcinoma: a systematic review and meta-analysis. Lancet Oncol. 23, 521–530. 10.1016/S1470-2045(22)00078-X 35255263 PMC9718369

[B75] Topel H. Bagirsakçi E. Yilmaz Y. Günes A. Bagci G. Çömez D. (2021). High glucose induced c-Met activation promotes aggressive phenotype and regulates expression of glucose metabolism genes in HCC cells. Sci. Rep. 11, 11376. 10.1038/s41598-021-89765-5 34059694 PMC8166976

[B76] Vander Heiden M. G. Cantley L. C. Thompson C. B. (2009). Understanding the Warburg effect: the metabolic requirements of cell proliferation. Science 324, 1029–1033. 10.1126/science.1160809 19460998 PMC2849637

[B77] Van Der Windt G. J. W. Everts B. Chang C. H. Curtis J. D. Freitas T. C. Amiel E. (2012). Mitochondrial respiratory capacity is a critical regulator of CD8+T cell memory development. Immunity 36, 68–78. 10.1016/j.immuni.2011.12.007 22206904 PMC3269311

[B78] Villanueva A. (2019). Hepatocellular carcinoma. N. Engl. J. Med. 380, 1450–1462. 10.1056/NEJMra1713263 30970190

[B79] Wang J. W. Maniruzzaman M. (2022). A global bibliometric and visualized analysis of bacteria-mediated cancer therapy. Drug Discov. Today 27, 103297. 10.1016/j.drudis.2022.05.023 35654388 PMC9530009

[B80] Wang S. L. Liu G. H. Li Y. R. Pan Y. B. (2022). Metabolic reprogramming induces macrophage polarization in the tumor microenvironment. Fron. Immunol. 13, 840029. 10.3389/fimmu.2022.840029 PMC9302576 35874739

[B81] Warburg O. (1956). On the origin of cancer cells. Science 123, 309–314. 10.1126/science.123.3191.309 13298683

[B82] Watson M. J. Vignali P. D. A. Mullett S. J. Overacre-Delgoffe A. E. Peralta R. M. Grebinoski S. (2021). Metabolic support of tumour-infiltrating regulatory T cells by lactic acid. Nature 591, 645–651. 10.1038/s41586-020-03045-2 33589820 PMC7990682

[B83] Wu J. Y. Huang T. W. Hsieh Y. T. Wang Y. F. Yen C. C. Lee G. L. (2020). Cancer-derived succinate promotes macrophage polarization and cancer metastasis via succinate receptor. Mol. Cell 77, 213–227. 10.1016/j.molcel.2019.10.023 31735641

[B84] Xia L. Oyang L. Lin J. Tan S. Han Y. Wu N. (2021). The cancer metabolic reprogramming and immune response. Mol. Cancer 20, 28. 10.1186/s12943-021-01316-8 33546704 PMC7863491

[B85] Xia Y. J. Brown Z. J. Huang H. Tsung A. (2021). Metabolic reprogramming of immune cells: shaping the tumor microenvironment in hepatocellular carcinoma. Cancer Med. 10, 6374–6383. 10.1002/cam4.4177 34390203 PMC8446566

[B86] Xu X. Peng Q. Jiang X. Tan S. Yang Y. Yang W. (2023). Metabolic reprogramming and epigenetic modifications in cancer: from the impacts and mechanisms to the treatment potential. Exp. Mol. Med. 55, 1357–1370. 10.1038/s12276-023-01020-1 37394582 PMC10394076

[B87] Yang Z. Yan C. Ma J. Peng P. Ren X. Cai S. (2023). Lactylome analysis suggests lactylation-dependent mechanisms of metabolic adaptation in hepatocellular carcinoma. Nat. Metab. 5, 61–79. 10.1038/s42255-022-00710-w 36593272

[B88] Zhang C. Hu J. J. Sheng L. Yuan M. Wu Y. Chen L. (2019). Metformin delays AKT/c-Met-driven hepatocarcinogenesis by regulating signaling pathways for *de novo* lipogenesis and ATP generation. Toxico. Appl. Pharmacol. 365, 51–60. 10.1016/j.taap.2019.01.004 30625338

[B89] Zhang C. H. Cheng Y. F. Zhang S. Fan J. Gao Q. (2022). Changing epidemiology of hepatocellular carcinoma in Asia. Liver Int. 42, 2029–2041. 10.1111/liv.15251 35319165

[B90] Zhang Q. Wang H. R. Mao C. Y. Sun M. Dominah G. Chen L. Y. (2018). Fatty acid oxidation contributes to IL-1β secretion in M2 macrophages and promotes macrophage-mediated tumor cell migration. Mol. Immunol. 94, 27–35. 10.1016/j.molimm.2017.12.011 29248877 PMC5801116

[B91] Zhang X. Coker O. O. Chu E. S. Fu K. Lau H. C. H. Wang Y. X. (2021). Dietary cholesterol drives fatty liver-associated liver cancer by modulating gut microbiota and metabolites. Gut 70, 761–774. 10.1136/gutjnl-2019-319664 32694178 PMC7948195

[B92] Zucman-Rossi J. Villanueva A. Nault J. C. Llovet J. M. (2015). Genetic landscape and biomarkers of hepatocellular carcinoma. Gastroenterology 149, 1226–1239. 10.1053/j.gastro.2015.05.061 26099527

