# Supplementary material for: Metabolic reprogramming in hepatocellular carcinoma: a bibliometric and visualized study from 2011 to 2023
Source: Front Pharmacol. 2024 Jul 16;15:1392241. doi: 10.3389/fphar.2024.1392241 (PMC11289777; doi:10.3389/fphar.2024.1392241)
Supplement: Supplementary file 1 [file Table1.DOCX]

**Table 1. Annual total citation per year.**

| **Year** | **MeanTCperArt** | **N** | **MeanTCperYear** | **CitableYears** |
| --- | --- | --- | --- | --- |
| 2011 | 84 | 2.00 | 6.46 | 13 |
| 2012 | 43 | 1.00 | 3.58 | 12 |
| 2013 | 66 | 3.00 | 6.00 | 11 |
| 2014 | 43.43 | 14.00 | 4.34 | 10 |
| 2015 | 87 | 18.00 | 9.67 | 9 |
| 2016 | 45.58 | 24.00 | 5.70 | 8 |
| 2017 | 86.44 | 32.00 | 12.35 | 7 |
| 2018 | 48.39 | 41.00 | 8.06 | 6 |
| 2019 | 46.48 | 52.00 | 9.30 | 5 |
| 2020 | 36.1 | 72.00 | 9.03 | 4 |
| 2021 | 17.31 | 97.00 | 5.77 | 3 |
| 2022 | 7.61 | 114.00 | 3.81 | 2 |
| 2023 | 1.46 | 105.00 | 1.46 | 1 |

MeanTCperArt, Mean of total citations per article. N, number of articles. MeanTCperYear, Mean of total citations per year.

**Table 2. Information about the top ten journals.**

| **Journal** | **Articles** | **TCs** | **H index** | **IF2022** | **JCR area** |
| --- | --- | --- | --- | --- | --- |
| Cancers | 29 | 609 | 10 | 5.2 | Q2 |
| Frontiers in Oncology | 20 | 373 | 7 | 4.7 | Q2 |
| International Journal of Molecular Sciences | 19 | 335 | 11 | 5.6 | Q1 |
| Hepatology | 16 | 606 | 10 | 13.5 | Q1 |
| Cancer Research | 13 | 410 | 8 | 11.2 | Q1 |
| Journal of Hepatology | 12 | 592 | 8 | 25.7 | Q1 |
| Cells | 11 | 236 | 7 | 6 | Q2 |
| Frontiers in Immunology | 11 | 18 | 2 | 7.3 | Q1 |
| Oncogene | 10 | 448 | 8 | 8 | Q1 |
| Cancer Letters | 9 | 384 | 8 | 9.7 | Q1 |

TCs, total citations. IF, impact factor.

**Table 3. The number of publications in the top ten countries.**

| **Country** | **Articles** | **SCP** | **MCP** | **Freq** | **MCP Ratio** |
| --- | --- | --- | --- | --- | --- |
| China | 335 | 289 | 46 | 0.583 | 0.137 |
| USA | 69 | 52 | 17 | 0.12 | 0.246 |
| Italy | 31 | 23 | 8 | 0.054 | 0.258 |
| France | 18 | 10 | 8 | 0.031 | 0.444 |
| Germany | 14 | 9 | 5 | 0.024 | 0.357 |
| Korea | 12 | 9 | 3 | 0.021 | 0.25 |
| Japan | 11 | 9 | 2 | 0.019 | 0.182 |
| Spain | 11 | 4 | 7 | 0.019 | 0.636 |
| Iran | 6 | 1 | 5 | 0.01 | 0.833 |
| India | 5 | 3 | 2 | 0.009 | 0.4 |

MCP, number of papers co-authored with authors from other countries. SCP, number of papers co-authored with authors from the same country. Freq, frequency, representing the degree of contribution to this field.

**Table 4. Average citations in the top 10 TC countries.**

| **Country** | **TC** | **Average Article Citations** |
| --- | --- | --- |
| China | 7791 | 23.30 |
| USA | 2139 | 31.00 |
| Italy | 1251 | 40.40 |
| France | 825 | 45.80 |
| Japan | 530 | 48.20 |
| Korea | 266 | 22.20 |
| Germany | 245 | 17.50 |
| Spain | 224 | 20.40 |
| Mexico | 189 | 47.20 |
| Switzerland | 189 | 37.80 |

**Table 5 Papers of higher importance.**

| **Paper** | **DOI** | **Year** | **LCS** | **GCS** | **Cluster** |
| --- | --- | --- | --- | --- | --- |
| ALLY A, 2017, CELL | 10.1016/J.CELL.2017.05.046 | 2017 | 22 | 1178 | 4 |
| GAO Q, 2019, CELL | 10.1016/J.CELL.2019.08.052 | 2019 | 20 | 404 | 4 |
| SENNI N, 2019, GUT | 10.1136/GUTJNL-2017-315448 | 2019 | 19 | 77 | 4 |
| SHANG RZ, 2016, WORLD J GASTROENTERO | 10.3748/WJG.V22.I45.9933 | 2016 | 16 | 74 | 1 |
| GUO WJ, 2015, HEPATOLOGY | 10.1002/HEP.27929 | 2015 | 16 | 180 | 2 |
| NAKAGAWA H, 2018, CANCERS | 10.3390/CANCERS10110447 | 2018 | 16 | 84 | 4 |
| CHEN CL, 2016, CELL METAB | 10.1016/J.CMET.2015.12.004 | 2016 | 15 | 249 | 4 |
| FUJIWARA N, 2018, GUT | 10.1136/GUTJNL-2017-315193 | 2018 | 14 | 100 | 4 |
| LI JB, 2015, J HEPATOL | 10.1016/J.JHEP.2015.07.039 | 2015 | 12 | 140 | 1 |
| LIU MX, 2018, ONCOGENE | 10.1038/S41388-017-0070-6 | 2018 | 12 | 102 | 3 |
| WONG CCL, 2014, PLOS ONE | 10.1371/JOURNAL.PONE.0115036 | 2014 | 10 | 50 | 1 |
| XU F, 2019, MOL THER-NUCL ACIDS | 10.1016/J.OMTN.2019.09.002 | 2019 | 10 | 48 | 2 |
| DU DY, 2022, ACTA PHARM SIN B | 10.1016/J.APSB.2021.09.019 | 2022 | 10 | 109 | 3 |
| SANGINETO M, 2020, CANCERS | 10.3390/CANCERS12061419 | 2020 | 10 | 72 | 4 |
| HUANG QC, 2014, J HEPATOL | 10.1016/J.JHEP.2014.04.035 | 2014 | 9 | 120 | 1 |
| SUN LC, 2015, CELL RES | 10.1038/CR.2015.33 | 2015 | 9 | 199 | 3 |
| TANG L, 2018, CLIN CANCER RES | 10.1158/1078-0432.CCR-17-1707 | 2018 | 9 | 78 | 4 |

LCS, local citations. GCS, global citations.
